# Multiple Substrate Usage of *Coxiella burnetii* to Feed a Bipartite Metabolic Network

**DOI:** 10.3389/fcimb.2017.00285

**Published:** 2017-06-29

**Authors:** Ina Häuslein, Franck Cantet, Sarah Reschke, Fan Chen, Matteo Bonazzi, Wolfgang Eisenreich

**Affiliations:** ^1^Department of Chemistry, Chair of Biochemistry, Technische Universität MünchenGarching, Germany; ^2^IRIM-UMR 9004, Infectious Disease Research Institute of Montpellier, Université de Montpellier, Centre National de la Recherche ScientifiqueMontpellier, France

**Keywords:** *Coxiella*, isotopolog profiling, metabolism, nutrition, shikimate pathway, bipartite metabolism, *Legionella*

## Abstract

The human pathogen *Coxiella burnetii* causes Q-fever and is classified as a category B bio-weapon. Exploiting the development of the axenic growth medium ACCM-2, we have now used ^13^C-labeling experiments and isotopolog profiling to investigate the highly diverse metabolic network of *C. burnetii*. To this aim, *C. burnetii* RSA 439 NMII was cultured in ACCM-2 containing 5 mM of either [U-^13^C_3_]serine, [U-^13^C_6_]glucose, or [U-^13^C_3_]glycerol until the late-logarithmic phase. GC/MS-based isotopolog profiling of protein-derived amino acids, methanol-soluble polar metabolites, fatty acids, and cell wall components (e.g., diaminopimelate and sugars) from the labeled bacteria revealed differential incorporation rates and isotopolog profiles. These data served to decipher the diverse usages of the labeled substrates and the relative carbon fluxes into the core metabolism of the pathogen. Whereas, *de novo* biosynthesis from any of these substrates could not be found for histidine, isoleucine, leucine, lysine, phenylalanine, proline and valine, the other amino acids and metabolites under study acquired ^13^C-label at specific rates depending on the nature of the tracer compound. Glucose was directly used for cell wall biosynthesis, but was also converted into pyruvate (and its downstream metabolites) through the glycolytic pathway or into erythrose 4-phosphate (e.g., for the biosynthesis of tyrosine) via the non-oxidative pentose phosphate pathway. Glycerol efficiently served as a gluconeogenetic substrate and could also be used via phosphoenolpyruvate and diaminopimelate as a major carbon source for cell wall biosynthesis. In contrast, exogenous serine was mainly utilized in downstream metabolic processes, e.g., via acetyl-CoA in a complete citrate cycle with fluxes in the oxidative direction and as a carbon feed for fatty acid biosynthesis. In summary, the data reflect multiple and differential substrate usages by *C. burnetii* in a bipartite-type metabolic network, resembling the overall topology of the related pathogen *Legionella pneumophila*. These strategies could benefit the metabolic capacities of the pathogens also as a trait to adapt for replication under intracellular conditions.

## Introduction

The obligate intracellular Gram negative bacterium *Coxiella burnetii* is the causative agent of Q-fever, a worldwide-distributed zoonosis, except New Zealand (Maurin and Raoult, 1999; Arricau-Bouvery and Rodolakis, 2005). The disease can cause acute or chronic infections when the bacteria invade alveolar macrophages or other phagocytic and non-phagocytic cells in different tissues and organs, finally leading to endocarditis and hepatitis in its chronical form (Van Schaik et al., 2013). *C. burnetii* represents a category B bio-weapon due to its remarkable low infectious dose, since one bacterial cell in the lung is enough to cause the acute infection (Fournier et al., 1998; Madariaga et al., 2003). In addition, *C. burnetii* can form specific small cell variants (SCVs) that are stable for long time periods in the environment, also under harsh conditions (Coleman et al., 2004, 2007). After having invaded their target cells by passive phagocytosis, *C. burnetii* establishes an acidic phagolysosome-like replication compartment called *Coxiella*-containing vacuole (CCV) (Larson et al., 2016). Vacuolar acidification triggers the transition from SCV into the replicative large-cell variant (LCV) (Coleman et al., 2004). Establishment of the CCV as well as intracellular replication of *C. burnetii* inside of this specific compartment depends on a type IVB secretion system (T4SS), a homolog of the Icm/Dot secretion system in *Legionella pneumophila*, a closely relative of *Coxiella* (Seshadri et al., 2003; Beare et al., 2011; Carey et al., 2011). In both pathogens, these analog secretion systems translocate numerous effector proteins into their host cells to trigger the establishment of the replicative niche, to avoid degradation and to get access to sufficient amounts of nutrients (Zhu et al., 2011; Segal, 2013; Moffatt et al., 2015; Martinez et al., 2016). To date, more than 300 effector proteins are known for *L. pneumophila* and there seem to be even more along the *Legionella* species (Burstein et al., 2016; Hofer, 2016), whereas only about 60 proteins have been identified as effectors for *C. burnetii* (Chen et al., 2010; Carey et al., 2011; Newton et al., 2013). Still, the function of most of these effector proteins are not known, especially for *C. burnetii* (Chen et al., 2010; Weber et al., 2013).

However, these two pathogens also feature some major differences. Beside the fact that only few of the effector proteins are conserved, also the characteristics of the two replicative vacuoles differ. While *L. pneumophila* is replicating in a neutral environment within a compartment that is predominantly derived from the endoplasmatic reticulum, *C. burnetii* requires acidic conditions in its phagolysosome-like replication compartment (Hubber and Roy, 2010; Weber et al., 2013). Moreover, the invading pathogens target very different host cells for their multiplication. While *L. pneumophila* is specialized for replication inside protozoa and only accidentally infects human alveolar macrophages, *C. burnetii* is adapted for the infection of numerous mammalian species and tissues (Babudieri, 1959; Weber et al., 2013; Larson et al., 2016).

By now, it is well known that *L. pneumophila* preferably uses amino acids, especially serine as carbon and energy source (Tesh and Miller, 1981; Tesh et al., 1983). Recent studies showed that in addition to amino acids, *Legionella* uses further substrates like glucose and glycerol in a bipartite metabolic network (Eylert et al., 2010; Häuslein et al., 2015). Thereby, glucose is predominantly metabolized via the Entner-Doudoroff pathway (ED pathway) and not via glycolytic reactions (Schunder et al., 2014). However, glycerol predominantly serves gluconeogenetic reactions and is shuffled into the pentose phosphate pathway (PPP) (Häuslein et al., 2015).

In contrast to *L. pneumophila*, carbon metabolism of *C. burnetii* has been poorly investigated, since the possibility to grow this intracellular pathogen in an axenic medium has been developed only recently (Omsland et al., 2009, 2011).

**Figure 1:**
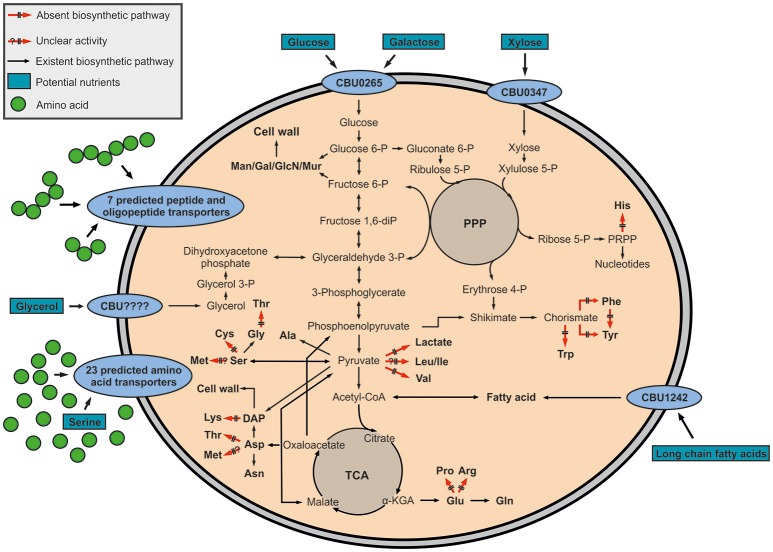
Hypothetical metabolic network of *Coxiella burnetii* RSA 439 NMII. The pathways are predicted on the basis of the genome sequence (Seshadri et al., 2003) and earlier biochemical studies (McDonald and Mallavia, 1971; Hackstadt and Williams, 1981a,b). Amino acids seem to be the main carbon and energy source of *Coxiella burnetii* RSA 439 NMII, since 23 amino acid transporters and 7 peptide and oligopeptide transporters have been predicted. Furthermore, some sugar transporters (CBU0265 and CBU0347) and a transporter for long chain fatty acids (CBU1242) have been identified, indicating that the metabolic potential of *C. burnetii* is versatile. In addition, enzymes responsible for glycerol metabolism (*glpK*: glycerol kinase, *glpD*: glycerol 3-phosphate dehydrogenase) are present in *C. burnetii*, although a glycerol transporter has not been identified yet. Black or red arrows represent amino acids prototrophies or auxotrophies. Potential carbon nutrients are indicated by boxes. α-KGA, α-ketoglutarate; Man, mannose; Gal, galactose; GlcN, glucosamine; Mur, muramic acid; PRPP, 5-phosphoribosyl diphosphate; PPP, pentose phosphate pathway; TCA, TCA cycle.

On the basis of its genome, the pathogen shows high metabolic capacities, especially when compared to other intracellular replicating bacteria. This is probably due to a relatively low genome reduction process in *C. burnetii*, since more than 89.1% of its genome encode proteins (Andersson and Kurland, 1998; Seshadri et al., 2003). Different to *L. pneumophila*, which predominately utilizes the ED pathway for glucose degradation, *C. burnetii* seems to use glycolytic reactions for glucose catabolism, although a classical hexokinase is missing in the glycolytic cascade on the basis of the genome sequence (McDonald and Mallavia, 1971; Hackstadt and Williams, 1981a,b). Nevertheless, hexokinase activity has been demonstrated in cell-free extracts (Paretsky et al., 1962). Also, the conversion of glucose 6-phosphate to 6-phosphogluconate and ribulose 5-phosphate has been detected, although genes encoding these reactions have not yet been annotated (Consigli and Paretsky, 1962; McDonald and Mallavia, 1970). Enzymes important for fatty acid or amino acid biosynthesis, synthesis of vitamins and nucleic acids, and a complete TCA cycle are also present in *Coxiella* (Seshadri et al., 2003). Otherwise, enzymes for the glyoxylate pathway have not been identified until now, indicating that *Coxiella* does not use fatty acids for energy generation in contrast to *Mycobacterium tuberculosis* (Schnappinger et al., 2003; Seshadri et al., 2003), although a putative transporter for long chain fatty acids (CBU1242) was assigned in the genome of *C. burnetii* (Kuley et al., 2015). Notably, the shikimate/chorismate pathway is also evident from the genome sequence although some late steps leading to the aromatic amino acids appear to be missing (Seshadri et al., 2003; Walter et al., 2014) (Figure 1).

Based on the prediction of numerous amino acid and peptide transporters, amino acids seem to be among the major carbon and energy source of *C. burnetii* (Sandoz et al., 2016b). Moreover, the predicted presence of two sugar transporters (CBU0265 and CBU0347) indicates the uptake and subsequent metabolism of sugars (Seshadri et al., 2003). Additionally, although no glycerol transporter has been predicted, enzymes responsible for glycerol degradation (*glpK:* glycerol kinase, *glpD:* glycerol 3-phosphate dehydrogenase) are present in *C. burnetii*, indicating that glycerol could also serve as a nutrient (Seshadri et al., 2003) (Figure 1). Compared to *Chlamydia* or *Rickettsia*, intracellular *Coxiella* seems unable to import ATP from its host, since an ATP/ADP exchanger has not been identified (Zomorodipour and Andersson, 1999; Miller and Thompson, 2002).

Despite considerable efforts in investigating the usage of some substrates like glutamate, pyruvate, succinate and glucose by *C. burnetii* (Hackstadt and Williams, 1981a, 1983), the nutritional diversity remains unknown. Nevertheless, the composition of the recently developed axenic medium is giving a welcome hint to the preferred metabolites of this pathogen, since it carries high amounts of peptides and amino acids. Furthermore, glucose is also present in ACCM-2 (Omsland et al., 2009, 2011), however not in ACCM-D (Sandoz et al., 2016a).

In recent years, isotopolog profiling has proven to be a powerful approach to describe substrate and pathway usages for a number of pathogens including *L. pneumophila* (Eylert et al., 2010; Schunder et al., 2014; Häuslein et al., 2015; Gillmaier et al., 2016; Kern et al., 2016). The method is based on ^13^C-incorporation experiments using completely or selectively ^13^C-labeled substrates such as glucose, serine or glycerol. Due to the isotopic labels, specific ^13^C-enrichments and isotopolog compositions are generated in downstream metabolic products (e.g., amino acids, fatty acids and sugars) which can be determined by gas chromatography/mass spectrometry (GC/MS). By comparison of these patterns, the differential substrate usage and the metabolic pathways can be reconstructed. Here, we have used isotopolog profiling approaches to investigate the highly diverse metabolic network of *C. burnetii*. For this purpose, we grew *C. burnetii* RSA 439 NMII in the axenic medium ACCM-2 in the presence of either 5 mM [U-^13^C_6_]glucose, [U-^13^C_3_]serine or [U-^13^C_3_]glycerol. Isotopolog profiling revealed that the pathogen is capable to efficiently use all of these substrates in a bipartite-type metabolic network.

## Materials and methods

### Bacteria strains and growth conditions

For each growth condition, Acidified Citrate Cysteine Medium 2 (ACCM-2, Table S1) was inoculated with *C. burnetii* RSA 439 NMII at 1 × 10^5^ genome equivalents GE/mL. Bacteria were grown in a humidified atmosphere of 5% CO_2_ and 2.5% O_2_ at 37°C for the indicated times. GE/mL were calculated using the PicoGreen assay according to manufacturers' instructions. Briefly, 50 μL of bacterial culture were mixed to 5 μL of 10% Triton X-100 in a well of a 96-wells microplate with black bottom and sides and incubated 10 min at room temperature on a plate shaker. PicoGreen was then added to the mixture and the plates were incubated at room temperature and in the dark for 10 min. Sample fluorescence was measured using a fluorescence microplate reader using filters for standard fluorescein wavelengths (excitation ~480 nm, emission ~520 nm). DNA concentrations were obtained by plotting the fluorescence readings on a standard curve obtained applying the same approach to a dilution of a plasmid of known concentration. Data were converted to GE/mL by dividing the DNA concentration by the mass of the *Coxiella* genome (2.2 fg).

### Labeling and growth experiments of *coxiella burnetii* RSA 439 NMII

[U-^13^C_3_]Serine, [U-^13^C_6_]glucose and [U-^13^C_3_]glycerol were purchased from Isotec/Sigma-Aldrich. 1 L ACCM-2 supplemented either with 5 M [U-^13^C_3_]serine, 5 mM [U-^13^C_6_]glucose or 5 mM [U-^13^C_3_]glycerol was inoculated with *C. burnetii* RSA 439 NMII as indicated above. Seven days post-inoculation, bacteria were harvested by centrifugation at 4.500 g for 45 min at 4°C. Bacterial pellet (ca. 10^14^ bacteria per condition) was autoclaved for 30 min at 120°C. Cells were freeze-dried and stored at −80°C until further analysis. To evaluate the impact of each compound on bacterial growth, ACCM-2 was supplemented with the corresponding unlabeled compounds at 5 mM and inoculated with *C. burnetii* RSA 439 NMII at 10^5^ GE/mL. Bacterial growth was assessed at 0, 3, 5, and 7 days post-inoculation using the PicoGreen assay as previously described (Martinez et al., 2014).

### Sample preparation and derivatization of protein-derived amino acids and cell wall-derived diaminopimelate (DAP)

For isotopolog profiling of protein-derived amino acids and cell wall-derived DAP, approximately 10^9^ bacterial cells (about 1 mg of freeze-dried pellet) were hydrolyzed in 0.5 mL of 6 M HCl for 24 h at 105°C, as described earlier (Eylert et al., 2010). Samples were dried under a stream of nitrogen at 70°C. The residue was solved in 200 μL of acetic acid and purified on a cation exchange column of Dowex 50WX8 (H+-form, 200–400 mesh, 5 × 10 mM), which was previously washed with 1 mL MeOH and 1 mL ddH_2_O. Elution occurred after washing with 2 mL of ddH_2_O with 1 mL of 4 M ammonium hydroxide. 200 μL of the eluate were dried at 70°C under a stream of nitrogen and dissolved in 50 μL dry acetonitrile and 50 μL *N*-(tert-butyldimethyl-silyl)-*N*-methyl-trifluoroacetamide containing 1% tert-butyl-dimethyl-silylchlorid (Sigma). Derivatization occurred at 70°C for 30 min. The resulting tert-butyl-dimethylsilyl (TBDMS) derivates of protein-derived amino acids and cell wall-derived DAP were analyzed by GC/MS. Due to degradation during acid hydrolysis, tryptophan, methionine and cysteine could not be analyzed with this method. Furthermore, acid hydrolysis leads to conversion of glutamine and asparagine to glutamate and aspartate, respectively. Therefore, results given for aspartate and glutamate correspond to asparagine/aspartate and glutamine/glutamate, respectively. Due to inefficient derivatization, TBDMS-arginine could not be detected in sufficient amounts for isotopolog profiling.

### Sample preparation and derivatization of methanol-soluble polar metabolites

For isotopolog profiling of methanol-soluble polar metabolites including fatty acids, approximately 5 mg of the freeze-dried bacteria were mixed with 0.8 g of glass beads (0.25–0.05 mM). 1 mL of pre-cooled 100% methanol was added and mechanical cell lysis occurred for 3 × 20 s at 6.5 m/s using a ribolyser instrument (Hybaid). Samples were immediately cooled down on ice for 5 min followed by centrifugation at 2.300 × g for 10 min. The supernatant was dried under a stream of nitrogen. The residue was dissolved in 50 μL dry acetonitrile and 50 μL *N*-(tert-butyldimethyl-silyl)-*N*-methyl-trifluoroacetamide containing 1% tert-butyl-dimethyl-silylchlorid (Sigma) and kept at 70°C for 30 min. The resulting tert-butyl-dimethylsilyl (TBDMS) derivates of methanol-soluble polar metabolites and fatty acids were used in GC/MS analysis.

### Sample preparation and derivatization of mannose (man) and galactose (gal)

For isotopolog profiling of Man and Gal, 5 mg of the freeze-dried bacteria were methanolized over night at 80°C with 0.5 mL methanolic HCl (3 M). Samples were cooled down and the supernatant was dried at 25°C under a stream of nitrogen. The first step of derivatization occurred at 25°C for 1 h in 1 mL acetone containing 20 μL concentrated H_2_SO_4_. After that, 2 mL of saturated NaCl and saturated Na_2_CO_3_ solution was added. The aqueous solution was extracted 2x with 3 mL ethyl acetate. The combined organic phases were dried under a stream of nitrogen. Dry residue was incubated overnight at 60°C with 200 μL of a 1:1 mixture of dry ethyl acetate and acetic anhydride in a second derivatization step. Derivatization reagents were removed under a stream of nitrogen and the remaining residue was resolved in 100 μL anhydrous ethyl acetate. Resulting diisopropylidene/acetate derivatives were used for GC/MS analysis.

### Sample preparation and derivatization of cell wall-derived glucosamine (GlcN) and muramic acid (mur)

For isotopolog profiling of GlcN and Mur, cell wall hydrolyzation was performed with approximately 15 mg of freeze-dried bacterial in 0.5 mL of 6 M HCl overnight at 105°C. Solid components were removed by filtration. The filtrate was dried under a stream of nitrogen and 100 μL of hexamethyldisilazane (HMDS) was added. Derivatization occurred for 3 h at 120°C. Resulting TMS-derivatives were used for GC/MS analysis.

### Gas chromatography/mass spectrometry

GC/MS-analysis was performed with a QP2010 Plus gas chromatograph/mass spectrometer (Shimadzu) equipped with a fused silica capillary column (Equity TM-5; 30 m × 0.25 mM, 0.25 μm film thickness; SUPELCO) and a quadrupole detector working with electron impact ionization at 70 eV. An aliquot (0.1 to 6 μL) of the derivatized samples were injected in 1:5 split mode at an interface temperature of 260°C and a helium inlet pressure of 70 kPa. Selected ion monitoring was used with a sampling rate of 0.5 s and LabSolution software (Shimadzu) was used for data collection and analysis. For technical replicates, samples were measured three times, respectively. Overall ^13^C-excess values and isotopolog compositions where calculated as described earlier (Eylert et al., 2008). This involved (i) determination of GC/MS spectra of unlabeled derivatized metabolites, (ii) determination of the absolute mass of isotopolog enrichments and distributions of labeled metabolites of the experiment and (iii) correction of the absolute ^13^C-incorporation by subtracting the heavy isotopolog contributions due to the natural abundances in the derivatized metabolites.

For amino acid and DAP analysis, the following temperature program was used: After sample injection, the column was first kept at 150°C for 3 min and then developed with a temperature gradient of 7°C min^−1^ to a final temperature of 280°C. This temperature was held for further 3 min. TBDMS-derivatives of alanine (6.7 min), glycine (7.0 min), valine (8.5 min), leucine (9.1 min), isoleucine (9.5 min), proline (10.1 min), serine (13.2 min), phenylalanine (14.5 min), aspartate (15.4 min), glutamate (16.8 min), lysine (18.1 min), histidine (20.4 min), tyrosine (21.0 min), and the cell wall component DAP (23.4 min) were detected and isotopolog calculations were performed with m/z [M-57]^+^ or m/z [M-85]^+^.

For analysis of methanol-soluble metabolites including palmitate and stearate, the column was kept at 100°C for 2 min after sample injection. This was followed by a first temperature gradient, in which the column was heated to a final temperature of 234°C in rates of 3°C min^−1^. Subsequently, a second temperature gradient of 1°C min^−1^ was used until a final temperature of 237°C, and a third temperature gradient of 3°C min^−1^ to a final temperature of 260°C. TBDMS-derivatives of lactate (17.8 min), succinate (27.5 min), fumarate (28.7 min), malate (39.1 min), palmitate (44.0 min) and stearate (49.4 min) were detected. Isotopolog calculations were performed with m/z [M-57]^+^.

For diisopropylidene/acetate derivatives of the hexoses, Man and Gal, the column was kept at 150°C for 3 min after sample injection followed by a first temperature gradient of 10°C min^−1^ to a final temperature of 220°C, and a second temperature gradient of 50°C min^−1^ to a final temperature of 280°C. The final temperature was held for further 3 min. isotopolog calculations were performed with a fragment which still contains all six C-atoms of the hexoses (m/z 287 [M-15]^+^).

For analysis of the TMS-derivatives of the cell wall components GlcN and Mur, the column was kept at 70°C for 5 min followed by a temperature gradient of 5°C min^−1^ to a final temperature of 310°C, which was then kept for an additional minute. isotopolog calculations were performed with m/z [M-452]^+^ and m/z [M-434]^+^. Retention times and mass fragments of derivatized metabolites that were used for all isotopolog calculations are summarized in Table S2.

## Results

### Axenic growth of *C. burnetii* is not affected by exogenous serine, glucose, and glycerol

To investigate the effects of additional amounts of serine, glucose or glycerol on the axenic growth of *C. burnetii*, growth curves were calculated from ACCM-2 cultures supplemented with 5 mM of each substrate and compared to the growth curves of *C. burnetii* growing in standard ACCM-2. Cell densities were determined by calculating the genome equivalents/mL (GE/mL) at 3, 5, and 7 days post inoculation. No significant effect on the growth rates of *C. burnetii* could be observed under this experimental setup (Figure S1). To study the usage of serine, glucose and glycerol by this pathogen, we subsequently performed labeling experiments with [U-^13^C_3_]serine, [U-^13^C_6_]glucose and [U-^13^C_3_]glycerol.

### Utilization of serine by *C. burnetii* growing in ACCM-2

**Figure 2:**
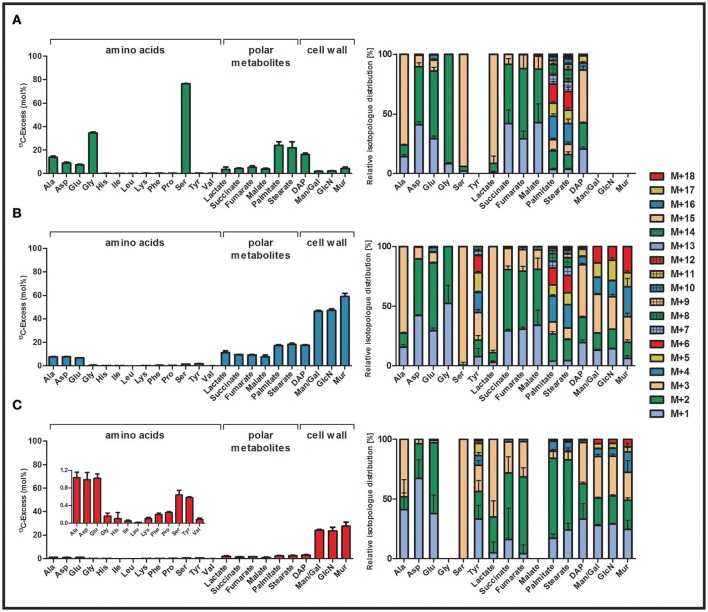
^13^C-Excess (mol%) and the fractional isotopolog distributions (%) in key metabolites of *Coxiella burnetii* RSA 439 NMII grown in an axenic ACCM-2 medium supplied with a ^13^C-labeled tracers. The bacteria were supplemented either with **(A)** 5 mM [U-^13^C_3_]serine, **(B)** 5 mM [U-^13^C_6_]glucose or **(C)** 5 mM [U-^13^C_3_]glycerol. The bacteria were harvested after 7 days of growth. ^13^C-Excess (mol%) and relative fractions of isotopologs (%) into protein-derived amino acids, methanol-soluble polar metabolites and cell wall components (DAP, Man/Gal, GlcN and Mur) were determined by GC/MS. Error bars indicate standard deviations from the means of six values (2 x biological replicates, 3 x technical GC/MS). For numerical values, see Tables S3 and S5. DAP, diaminopimelate; Man, mannose; Gal, galactose; GlcN, glucosamine; Mur, muramic acid.

To study serine metabolism in *C. burnetii*, we performed labeling experiments in ACCM-2 supplemented with 5 mM [U-^13^C_3_]serine. ^13^C-Enrichments and isotopolog fractions of protein-derived amino acids, polar metabolites, fatty acids, DAP and amino sugars derived from cell wall biosynthesis were measured by GC/MS. No ^13^C-incorporation occurred into His, Ile, Leu, Lys, Phe, Pro, Tyr, and Val that are obviously taken in unlabeled form from the respective amino acids and peptides present in the axenic medium. Only small fractions of ^13^C-label (<5%) could be found in sugars (from cell wall hydrolysates). In sharp contrast, very high ^13^C-enrichments were detected in Ser (76.3%) and Gly (34.6%) from the cell hydrolysates. This indicates that exogenous Ser was efficiently taken up from the axenic medium and might be converted into Gly by serine hydroxymethyltransferase (CBU1419). Furthermore, metabolites due to Ser conversion into pyruvate, acetyl-CoA, intermediates of the TCA cycle and their downstream products showed high ^13^C-enrichments (palmitate: 24.0% > stearate: 21.8% > DAP: 16.3% > Ala: 13.8% > Asp: 9.0% > Glu: 7.5% > fumarate: 5.2% > succinate: 4.4% > malate: 3.8%) (Figure 2A, Tables S3 and S5). On the basis of the fractional isotopolog abundances, a more detailed analysis of the metabolic pathways affording the observed patterns (for numerical values, see Table S3) was now done.

#### Ser and gly

Ser from the hydrolysate was mainly found as the ^13^C_3_- isotopolog (i.e., carrying three ^13^C-labels; M+3 in Figure 2A, Table S5). However, Ser also showed minor amounts of M+2 (i.e., the ^13^C_2_- isotopolog) as an indication of metabolic turnover (i.e., degradation to e.g., M+2 Gly and resynthesis of Ser finally leading to M+2 Ser). This is confirmed by the detected isotopolog profile of Gly characterized by the M+2 species.

#### Ala

High enrichment values in Ala (13.8%) with predominantly M+3 label reflect the formation of its precursor pyruvate from fully labeled M+3 Ser via serine dehydratase (CBU0194). Minor fractions of M+2 and M+1 in Ala also indicate the biosynthesis of this amino acid from M+2 or M+1 labeled pyruvate. This could be explained by pyruvate formation from M+1 and M+2 malate through the action of the malate dehydrogenase (oxaloacetate decarboxylating, CBU0823). Alternatively, or in addition, reactions via the phosphoenolpyruvate carboxykinase (CBU2092) could result in M+2 or M+1 label in phosphoenolpyruvate and subsequently in pyruvate due to reactions via pyruvate kinase (CBU1781).

#### Succinate, fumarate, malate, asp, glu

Asp (9.0%) directly derived from oxaloacetate and Glu (7.5%) synthesized from α-ketoglutarate were highly ^13^C-enriched. High levels of M+2 fractions, especially in Glu, are in line with a TCA cycle operating in the oxidative direction, starting from ^13^C_2_-acetyl-CoA. Assuming *Si*-specificity of the citrate synthase reaction, both labels from acetyl-CoA are retained in position 4 and 5 of α-ketoglutarate and its product Glu. On the basis of the sequence in the *C. burnetii* genome, the citrate synthase (CBU1410) is indeed expected to be *Si*-specific. Further reaction of [4,5-^13^C_2_]α-ketoglutarate via the TCA cycle then results in the detected [1,2-^13^C_2_]succinate, [1,2-^13^C_2_]fumarate, and [1,2-^13^C_2_]malate. [1,2-^13^C_2_]oxaloacetate is reflected by the observed [1,2-^13^C_2_]Asp isotopolog. It should be noted that the intrinsic symmetry of succinate randomizes the M+2 label between positions 1,2 and 3,4 finally also leading to [3,4-^13^C_2_]isotopologs in malate, oxaloacetate and Asp. The detected minor amounts of M+1 label could then easily be explained by multiple cycling in the TCA. Notably, significant amounts of M+3 isotopologs were also detected in Glu and Asp probably due to ^13^C-carbon flux from fully labeled pyruvate into M+3 malate via the malate dehydrogenase enzyme (malic enzyme, CBU0823).

#### Fatty acids

Palmitate (24.0%) and stearate (21.8%) were highly enriched, again indicating a high carbon flux from the labeled Ser tracer into [^13^C_2_]acetyl-CoA. Indeed, the isotopolog profiles of both fatty acids mainly showed M+2 or a multiple of M+2 due to the combination of fully labeled acetyl-CoA building blocks in fatty acid biosynthesis.

#### DAP

The biosynthesis of the cell wall component DAP, which is also an intermediate for lysine biosynthesis, depends on pyruvate and Asp as precursors. In contrast to Lys, where no ^13^C-enrichment was detectable, DAP showed high ^13^C-enrichment (16.3%). This indicates highly active enzymes to form DAP for cell wall formation, but not for Lys biosynthesis. This is remarkable since only one additional step catalyzed by diaminopimelate decarboxylase would be required to form Lys from DAP. Isotopolog distribution in DAP from the experiment with [U-^13^C_3_]serine mainly showed M+3 label, which is easily explained by the usage of M+3 labeled pyruvate (see above). Furthermore, some M+2 and M+1 fractions in DAP were also detected, probably due to M+2 and M+1 labeled Asp as a building unit (see above). Isotopologs carrying more than three ^13^C-atoms may be due to statistical combinations of labeled pyruvate and Asp.

#### Lactate

Interestingly, also lactate showed some minor ^13^C-enrichment in the experiment with labeled serine (Figure 2A). However, enzymes responsible for lactate biosynthesis from pyruvate seem not to be present in *C. burnetii* based on the genome sequence (Seshadri et al., 2003; Walter et al., 2014). Hypothetically, lactate could be produced from methylglyoxal due to detoxification reactions in this pathogen (Figure S2, for more details, see also below).

### Utilization of glucose

Uptake and incorporation of exogenous glucose were investigated by growth experiments in ACCM-2 supplemented with 5 mM [U-^13^C_6_]glucose. Workup and isotope analysis of metabolites was done as described above for the labeling experiments with ^13^C-Ser. High ^13^C-excess values (40–50%) could be detected in cell wall sugars indicating that the exogenous glucose tracer was efficiently taken up and metabolized (Figure 2B). On this basis, it is obvious that the pathogen can use glucose as a main carbon substrate under the experimental conditions. More specifically, significant ^13^C-enrichments from [U-^13^C_6_]glucose were detected in muramic acid (Mur: 59.1%) > glucosamine (GlcN: 47.2%) > mannose/galactose (Man/Gal: 46.3%) > diaminopimelate (DAP: 17.5%) as well as in fatty acids (stearate: 18.3% > palmitate: 17.3%) and metabolites related to pyruvate biosynthesis and reactions in the TCA cycle (succinate: 9.3% > fumarate: 9.2% > Asp: 7.8% > Ala: 7.7% > malate: 7.5% > Glu: 6.8%). Additionally, low but significant ^13^C-excess values were detected in Ser (1.4%) and Gly (0.5%), which clearly shows some *de novo* biosynthesis of Ser directly from a fully labeled C_3_-precursor (pyruvate or 3-phosphoglycerate). Labeled Ser is then directly converted into Gly. This is also reflected by the appearance of almost exclusive M+3 label in Ser and M+2 label in Gly. Low, but significant enrichments could be found in Tyr (1.7%), an amino acid derived from the shikimate pathway. No *de novo* biosynthesis could again be found for His, Ile, Leu, Lys, Phe, Pro and Val (Figure 2B, Tables S3 and S6).

Compared to the labeling experiment with [U-^13^C_3_]serine, Ala showed lower ^13^C-enrichments in the experiment with [U-^13^C_6_]glucose. However, similar overall ^13^C-enrichment values appeared in Asp and Glu as well as in fatty acids and DAP in both labeling experiments. This indicates that *C. burnetii* uses glucose as a carbon and energy source at similar rates as Ser. However, glucose is used more efficiently in the formation of amino sugars for cell wall biosynthesis directly or via glycolytic cycling, and serves as a precursor for the *de novo* synthesis of Ser, Gly and Tyr. On the contrary, Ser is almost exclusively metabolized in the TCA cycle for energy generation (Figures 2A,B). Like in the labeling experiments with ^13^C-Ser, also lactate sowed significant ^13^C-excess values (11.3%). The labeled lactate was again characterized by the M+3 isotopolog although enzymes for the direct biosynthesis via pyruvate are not annotated in *C. burnetii*. However, it can be assumed that this labeling appears due to detoxification reactions via methylglyoxal, which can be formed non-enzymatically from intermediates of glycolytic and gluconeogenetic reactions (Riddle and Lorenz, 1973; Omsland and Heinzen, 2011) (Figure S2). Furthermore, *C. burnetii* features a methylglyoxal synthase (CBU0853), which could catalyze the formation of methylglyoxal from dihydroxyacetone phosphate. The appearance of high amounts of methylglyoxal can therefore be related to high levels of glyceraldehyde 3-phosphate and dihydroxyacetone phosphate. Nevertheless, since methylglyoxal is toxic it has to be degraded via the glyoxalase system resulting in labeled lactate (Booth et al., 2003). However, *C. burnetii* lacks one enzyme of this detoxification system (glyoxalase I) (Seshadri et al., 2003; Omsland and Heinzen, 2011). The slow growth of this intracellular pathogen could therefore be due to avoiding the formation of high levels of methylglyoxal.

#### Sugars

The highly ^13^C-enriched amino sugars GlcN and Mur, which are directly derived from cell wall biosynthesis as well as Man/Gal predominantly showed M+3 and M+6 label. M+6 isotopologs indicate the direct usage of fully labeled ^13^C-glucose for cell wall biosynthesis, whereas M+3 suggests that glucose is efficiently used in glycolytic reactions to form fully labeled triose phosphates and related C_3_-metabolites. The latter ones can recombine with unlabeled C_3_-metabolites in gluconeogenetic reactions to form M+3 in Man/Gal and M+3 in GlcN and Mur for cell wall biosynthesis. M+2 label in these compounds could be explained by fully labeled acetyl-CoA entering the TCA cycle generating M+2 malate that could result in M+2 pyruvate by reaction of the malate dehydrogenase enzyme (malic enzyme, CBU0823). Gluconeogenetic reactions could then incorporate the M+2 label into the cell wall sugars. M+4 and M+5 isotopologs might result from combination of two labeled C_3_-precursors in gluconeogenetic reactions (Figure 2B and Table S6).

#### Fatty acids

Beside cell wall sugars, also fatty acids carried high amounts of ^13^C-label demonstrating the efficient conversion of [U-^13^C_6_]glucose into fully labeled acetyl-CoA which is subsequently used in *de novo* biosynthesis of fatty acids. This is also represented by the isotopolog patterns, since predominantly M+2 or a multiple of M+2 label was detected in palmitate and stearate (Figure 2B and Table S6).

#### Amino acids and polar metabolites

High rates of carbon flux from glucose via pyruvate and acetyl-CoA is also reflected by the ^13^C-enrichments of Ala (7.7%), succinate (9.4%), fumarate (9.2%), malate (7.6%), Asp (7.8%) and Glu (6.8%) (Figure 2B). High amounts of M+2 isotopologs in these metabolites are related to reactions in the TCA cycle. This clearly showed the usage of fully labeled acetyl-CoA, i.e., M+2, as a precursor, which is combined with oxaloacetate to form M+2 citrate and its M+2 downstream products α-ketoglutarate/Glu, succinate, fumarate, malate, and oxaloacetate/Asp, respectively. Furthermore, M+3 label in succinate, fumarate and malate, but also in Glu and Asp demonstrate the direct carbon flux of fully labeled C_3_-precursors (as seen in M+3 pyruvate/Ala) into reactions of the TCA cycle via the malic enzyme (CBU0823), which can interconvert malate and pyruvate (Figure 2B).

#### DAP

High values of ^13^C-enrichment in the cell wall component DAP (17.5%) result from high carbon flux of [U-^13^C_6_]glucose into its precursors pyruvate and Asp. In accordance, DAP predominantly showed M+3 label, indicating that fully labeled pyruvate is efficiently used for the biosynthesis of this compound. Nevertheless, also high quantities of M+2 and M+1 label were detectable in DAP, originating from M+2 and M+1 label in Asp. Additionally, also M+5 isotopologs of DAP were detectable in significant amounts, which is due to a combination reaction of fully labeled pyruvate and Asp carrying M+2 label. Similar to the experiments with ^13^C-Ser, no ^13^C-label was detectable in Lys, again demonstrating that diaminopimelate decarboxylase, which is responsible for the biosynthesis of this amino acid via DAP, is not present or active in *C. burnetii* (Figure 2B).

#### Tyr

**Figure 3:**
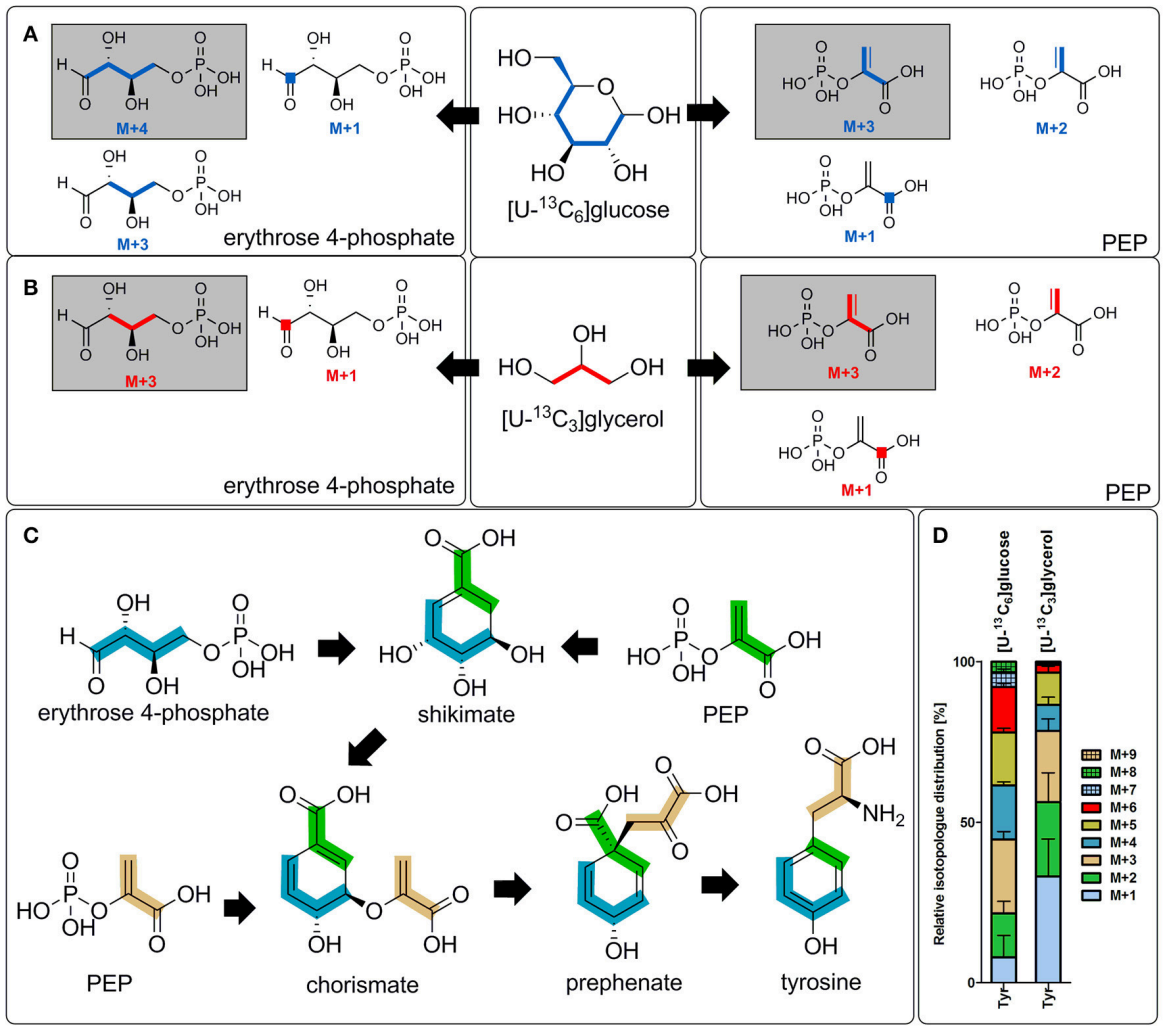
Transfer of ^13^C-label into tyrosine and its precursors. Transfer of ^13^C-label from [U-^13^C_6_]glucose **(A)** or [U-^13^C_3_]glycerol **(B)** into erythrose 4-phosphate (left) and PEP (right) due to distinct metabolic reactions in *C. burnetii*. This includes reactions of the pentose phosphate pathway (transketolase and transaldolase) as well as glycolytic and gluconeogenetic reactions. The respective isotopologs with the highest occurrence are highlighted (gray background). **(C)** Scheme of the biosynthesis of tyrosine in *C. burnetii*. The nine carbon atoms of tyrosine are derived from one molecule of erythrose 4-phosphate (four carbon atoms marked in blue) and two PEP molecules (marked in green and bright orange). Due to a final decarboxylation reaction, one carbon atom of the first PEP molecule (green) becomes lost. **(D)** Detected isotopolog composition of Tyr in the labeling experiment with [U-^13^C_6_]glucose or [U-^13^C_3_]glycerol, respectively. M+X, M represents the mass of the unlabeled metabolite plus X labeled ^13^C-atoms. PEP, phosphoenolpyruvate.

Low, but significant ^13^C-enrichments were detectable in Tyr (1.8%), an aromatic amino acid derived from the shikimate pathway. Shikimate is made by combination of phosphoenolpyruvate, which is built in glycolytic and gluconeogenetic reactions, and erythrose 4-phosphate, which is a key metabolite of the PPP. Subsequently, formation of chorismate occurs by reaction of shikimate with one more molecule of phosphoenolpyruvate (Figure 3C). Biosynthesis of tyrosine then occurs either directly from chorismate or via phenylalanine depending on the metabolic potential of the respective organism (Figure 1). Since no enrichment was detectable in phenylalanine in the labeling experiments of *C. burnetii* with ^13^C-glucose (Figure 2B), it can be concluded that this pathogen is auxotrophic for phenylalanine, but nevertheless can use the shikimate/chorismate pathway for direct biosynthesis of tyrosine.

The isotopolog profile of Tyr showed a complex mixture of M+1 to M+8 isotopologs (Figure 3D). The high amounts of M+3 in Tyr can be explained by the incorporation of M+3 PEP. Since one of the PEP precursors is decarboxylated during the biosynthetic pathway, the high fractions of M+2 labeling are also obvious (Figure 3). The high fraction of M+4 in Tyr could reflect erythrose 4-phosphate formation from fully labeled M+6 fructose 6-phosphate via the transketolase reactions. Furthermore, M+3 label in erythrose 4-phosphate can occur due to glycolytic reactions that form fully labeled glyceraldehyde 3-phosphate which is subsequently entering the PPP. Labeled glyceraldehyde 3-phosphate can also be used in gluconeogenetic reactions to form M+3 isotopologs of fructose which is subsequently leading to M+3 or M+1 labeled erythrose 4-phosphate due to further transketolase conversions (Figure 3A). Higher labeling (M+5 to M+8) is a result from various combination reactions from differently labeled C_4_ and C_3_ precursors in Tyr biosynthesis (Figure 3).

### Utilization of glycerol

To investigate whether *C. burnetii* can use further carbon sources besides Ser and glucose, labeling experiments were performed in ACCM-2 supplemented with 5 mM [U-^13^C_3_]glycerol. Bacteria were again harvested 7 days post inoculation and ^13^C-enrichments and isotopolog profiles in key metabolites were determined by GC/MS. High ^13^C-excess values were predominantly found in the cell wall sugars Mur (27.5%), GlcN (23.5%) as well as in Man/Gal (24.1%). The cell wall component DAP however, acquired ^13^C-label at 3.1% (Figure 2C).

Some ^13^C-enrichments were also detected in fatty acids (stearate: 2.6%; palmitate: 2.6%). Additionally, minor labeling was found in Ala, Asp, Glu, Ser and Tyr (1.0–0.6%) as well as in intermediates of the TCA cycle (1.6–1.0%). Furthermore, lactate (1.9%) showed low but significant enrichment. No carbon flux occurred into Gly, His, Ile, Leu, Lys, Phe, Pro and Val (Figure 2C, Tables S3 and S7).

#### Sugars

Since hexoses and amino sugars derived from the cell wall showed by far the highest ^13^C-incorporation (twenty-fold more ^13^C-enrichment than in amino acids related to the TCA cycle), glycerol is predominantly used by *C. burnetii* for gluconeogenetic reactions obviously to provide precursors for cell wall biosynthesis. This is also represented by the isotopolog profiles of the sugars under study, all of which mainly showed M+3 indicating the direct usage of fully labeled ^13^C_3_-precursors. Furthermore, the formation of M+6 isotopologs in these sugars could only occur if two fully labeled ^13^C_3_-precursors are combined, again representing the high carbon flux from [U-^13^C_3_]glycerol into gluconeogenetic reactions and cell wall biosynthesis (Figure 2C).

#### Fatty acids and TCA cycle-derived intermediates and products

Fatty acids like palmitate and stearate as well as amino acids related to the TCA cycle showed minor ^13^C-enrichments. On this basis, it can be assumed that some labeled glycerol was also directed toward the biosynthesis of acetyl-CoA, which is subsequently used in biosynthetic formation of fatty acids or in the TCA cycle for energy generation, respectively. This is also represented by the isotopolog profiles of these metabolites, mainly displaying the M+2 molecules. Fatty acids additionally showed low amounts of M+4 isotopologs due to combination of two fully labeled acetyl-CoA precursors. However, no isotopologs carrying more than four ^13^C-atoms were detectable in contrast to the experiments with ^13^C-Ser and ^13^C-glucose, again illustrating the lower carbon flux from glycerol toward the TCA cycle. Similar to the previously discussed labeling experiments, M+3 label occurred in intermediates of the TCA cycle due to reactions of the malic enzyme, which can transfer M+3 label from pyruvate into malate and subsequently into oxaloacetate (Figure 2C).

#### Ser and tyr

Low, but significant enrichment in Ser with exclusive M+3 label again shows *de novo* biosynthesis of this amino acids via fully labeled C_3_-precursors in *C. burnetii*. Interestingly, glycerol, like glucose, was also used in the shikimate pathway for some *de novo* biosynthesis of Tyr (Figures 2C,3D). The predominant M+3 label could be explained by the usage of M+3 phosphoenolpyruvate and/or M+3 erythrose 4-phosphate in the biosynthetic pathway as described above for the glucose experiment (Figures 3B,C).

#### DAP

Compared to the ^13^C-enrichments in further amino acids, relatively high amounts of ^13^C were found in the cell wall component DAP (3.1%). Thereby, M+1, M+2, and M+3 were the highest fractions in this metabolite. This was different to the labeling experiments with Ser and glucose, where also M+4 and M+5 isotopologs were observed. The high amounts of M+3 isotopologs are directly derived from fully labeled pyruvate whereas M+2 and M+1 label is explained from Asp due to M+2 and M+1 labeling in oxaloacetate. Since no M+4 and M+5 isotopologs were detected, it can be assumed that no combination reaction with labeled pyruvate and labeled Asp occurred in this experiment. Again, no enrichment was detectable in Lys due to the missing diaminopimelate decarboxylase in *C. burnetii*. (Figure 2C).

### Differential substrate usage of *C. burnetii*

To compare the relative contributions of serine, glucose and glycerol as carbon nutrients for *C. burnetii*, we have used concentrations of 5 mM for each of the labeled compounds as supplements to the medium. However, it can be expected that minor amounts of these compounds in unlabeled form are present in ACMM-2. Therefore, we have estimated these concentrations on the basis of the published data (Sales et al., 1995; Omsland et al., 2011; Sandoz et al., 2016a). Glycerol does not seem to be present in ACMM-2 and the concentrations of unlabeled serine and glucose are reported as 1.68 mM and 1.39 mM, respectively. Taking into account the amounts of unlabeled serine and glucose, the incorporation rates for serine and glucose can be estimated as ^13^C-excess (determined in the experiment with [U-^13^C_3_]serine) × 1.34 or ^13^C-excess (determined in the experiment with [U-^13^C_6_]glucose) × 1.27 (Table S4, Figure S3).

**Figure 4:**
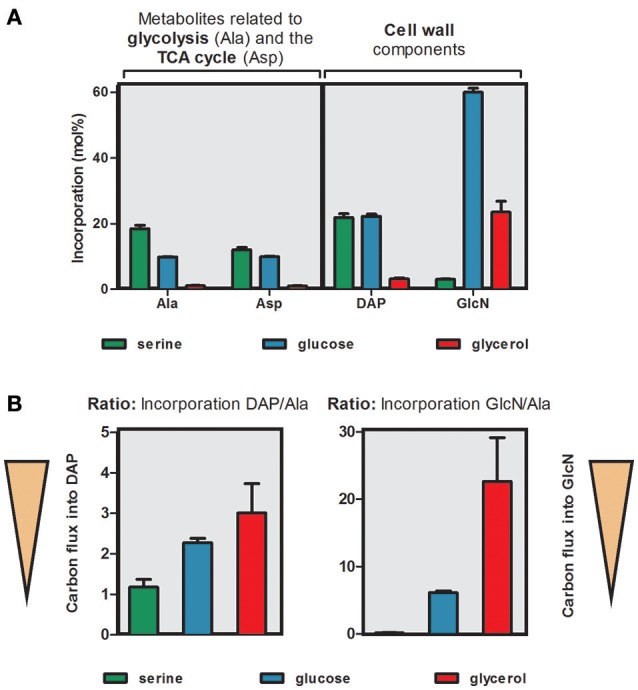
Differential usage of carbon substrates by *C. burnetii*. **(A)** ^13^C-Incorporation (mol%) into key metabolites related to carbon flux via glycolysis (Ala), the TCA cycle (Asp) or into cell wall derived components (DAP or GlcN). *C. burnetii* RSA 439 NMII wild-type was grown in ACCM-2 supplemented either with 5 mM [U-^13^C_3_]serine, 5 mM [U-^13^C_6_]glucose or 5 mM [U-^13^C_3_]glycerol. For numerical values, see Tables S3, S4. **(B)** Relative carbon flux from [U-^13^C_3_]serine, [U-^13^C_6_]glucose or [U-^13^C_3_]glycerol calculated from the ratio of ^13^C-excess values of key metabolites related to glycolysis (Ala) or the TCA cycle (Asp) in relation to ^13^C-excess values from cell wall components (DAP and GlcN). High values indicate that carbon flux is directed toward cell wall biosynthesis whereas low values indicate a high flux toward the TCA cycle.

To illustrate the differential carbon flux from the three labeled precursors [U-^13^C_3_]serine, [U-^13^C_6_]glucose and [U-^13^C_3_]glycerol, we selected four metabolic markers that are characteristic for specific carbon fluxes toward distinct metabolic pathways. Specifically, Ala was chosen as a marker metabolite for pyruvate biosynthesis and carbon flux toward e.g., the TCA cycle. Asp reflects a marker metabolite for the TCA cycle since it is directly derived from oxaloacetate, DAP is a marker metabolite for cell wall biosynthesis and finally GlcN represents a central cell wall component which is built from sugars including their formation via gluconeogenetic reactions. The %-incorporation values into these marker metabolites are compared in Figure 4A.

When grown with 5 mM ^13^C-Ser as a tracer, only marker metabolites related to ^13^C-carbon flux directed toward the TCA cycle display high ^13^C-incoroporation, whereas only very low ^13^C-incorporation was detectable in GlcN. In contrast, predominantly the amino sugar GlcN derived from gluconeogenetic reactions and cell wall biosynthesis reveal high enrichment values in the experiment with 5 mM ^13^C-glycerol, whereas Ala and Asp showed lower ^13^C-excess values. Nevertheless, also the cell wall component DAP showed higher incorporation values compared to other metabolites derived from the TCA cycle in the experiment with [U-^13^C_3_]glycerol. The labeling experiments with 5 mM ^13^C-glucose revealed high enrichment values in both groups (TCA cycle related markers and cell wall components) (Figure 4A). It turns out that serine and glucose are used as carbon nutrients at similar rates for feeding the glycolytic pathway providing pyruvate (for example, as a precursor for Ala and DAP) and acetyl-CoA (as a precursor for fatty acids and as a substrate for the TCA cycle). A major fraction of glucose is directly shuffled into hexose precursors for cell wall biosynthesis, as evident from the high incorporation rate into glucosamine. Glycerol, however, is rarely utilized as a carbon substrate for feeding glycolysis or the TCA, but is mainly used as a glucogenic source to fill up the pool of hexose phosphates required in cell wall biosynthesis, e.g., glucosamine (i.e., when glucose is not present at high concentrations in the environment). Interestingly, serine cannot serve as an efficient glucogenic substrate even at low glucose concentrations in the medium, as gleaned from the low incorporation of ^13^C-serine into glucosamine (Figure 4A).

To better display the relative carbon fluxes from a given substrate toward pyruvate biosynthesis, the TCA cycle and cell wall components, we now calculated the ratios of the ^13^C-excess values in DAP and GlcN over the ^13^C-excess values of Ala (Tables S8 and S9). High values indicate that ^13^C-carbon flux is directed toward cell wall biosynthesis e.g., of GlcN or DAP. Low values reflect high ^13^C-carbon fluxes into the TCA cycle e.g., for energy generation.

In the experiment with [U-^13^C_3_]serine as a precursor, the lowest values for both of these ratios were obtained, indicating the preferential usage of serine in the TCA cycle for energy generation by *C. burnetii*. Especially, the ratio “^13^C-excess GlcN/Ala” was very low, again reflecting the missing carbon flux from Ser into gluconeogenetic reactions and biosynthesis of GlcN. Minor carbon flux from Ser into DAP via the TCA cycle is also evident. In contrast, highest values for both ratios (^13^C-excess DAP/Ala and ^13^C-excess GlcN/Ala) were obtained when [U-^13^C_3_]glycerol was used as precursor. Therefore, this precursor seems to be especially fed into gluconeogenetic reactions for biosynthesis of cell wall sugars. However, glycerol is also shuffled into the TCA cycle albeit at lower rates, again predominantly serving the biosynthesis of the cell wall component DAP. Ratios were also high when [U-^13^C_6_]glucose was used as a precursor but did not reach the levels in the experiment with labeled glycerol. However, also glucose seems to be shuffled prevalently into cell wall biosynthesis directly or via glycolytic turnover (i.e., degradation by glycolysis and gluconeogenesis from C_3_-substrates). Moreover, the ratio “^13^C-excess DAP/Ala” was higher in this labeling experiment compared to that with ^13^C-Ser as a precursor. This indicates that glucose is also preferred for DAP biosynthesis although carbon flux into the TCA cycle seems to be similar in the experiments with glucose and Ser (Figure 4B).

In summary, these results show that carbohydrates and predominantly glycerol are preferred substrates of *C. burnetii* for the formation of cell wall components either directly using exogenous sugars or via gluconeogenetic reactions and via the TCA cycle to synthesize DAP. In contrast, serine serves as a substrate which is predominantly shuffled into the TCA cycle for energy generation and does not serve gluconeogenetic reactions efficiently. These findings support the model of a bipartite metabolic network which is discussed below.

## Discussion

In contrast to the genomes of other strictly intracellular bacteria, such as *Chlamydia trachomatis* or *Rickettsia*, the genome of *C. burnetii* reflects a high degree of metabolic capacities in a highly interconnected metabolic network with the potential usage of a single carbon substrate. On the other hand, its capacity to grow extracellularly in minimal media is restricted and indicates multiple substrate usages as described before for several intracellular bacteria (Grubmüller et al., 2014; Häuslein et al., 2015; Mehlitz et al., 2016). Here, we indeed show usage of exogenous serine, glucose and glycerol by *C. burnetii* during axenic growth with these substrates feeding specific sections of a partite metabolic network.

Incorporation rates and isotopolog profiles of amino acids, fatty acids, sugars and further metabolic intermediates demonstrate that glucose is efficiently taken up and utilized by *C. burnetii*, however, glucose alone does not support axenic growth of this pathogen in ACCM-2 (Figure S1) or in a minimal defined medium (ACCM-D) (Sandoz et al., 2016a). Furthermore, a classical hexokinase has not been identified in the genome of *C. burnetii*. The virtual absence of this essential enzymatic reaction for glucose catabolism could be by-passed by enzyme I of the bacterial phosphoenolpyruvate: sugar phosphotransferase system (PTS, CBU1550) and the histidine phosphocarrier protein (HPr, CBU0743) which are both present in *C. burnetii* (Seshadri et al., 2003). In line with our findings, differential gene expression studies also suggest that glucose is among the major substrates under *in vivo* conditions (Kuley et al., 2015). Our data confirm that *C. burnetii* can use glucose directly for the biosynthesis of cell wall compounds, but also to generate energy via glycolysis and the TCA cycle. This metabolic strategy is different to that of its close relative *L. pneumophila*, which mainly uses amino acids as energy source, as a possible strategy to limit the metabolic stress on the host cell, since disturbed glucose levels in eukaryotic cells are directly linked to apoptosis (Zhao et al., 2008). Considering that growth of *C. burnetii* is slower than that of *L. pneumophila*, a continuous low-rate usage of glucose by intracellular *C. burnetii* could therefore be possible without a further disturbance of host cell regulatory effects.

The labeling data show, for the first time, the effective uptake and usage of glycerol by *C. burnetii*. This provides direct evidence that, besides amino acids and glucose, further carbon sources not present in the currently used axenic media can comprise nutritional factors in the growth of this pathogen. Despite the identification of genes encoding enzymes involved in the catabolism of glycerol in the genome of *C. burnetii*, no glycerol transporter has been identified until now (Seshadri et al., 2003). Nevertheless, incorporation of this nutrient could also occur via passive diffusion through the bacterial membrane (Romijn et al., 1972; McElhaney et al., 1973). Our data now show that glycerol is preferably used in gluconeogenetic reactions, thereby predominantly serving the biosynthesis of peptidoglycan building units, like GlcN and Mur. A minor fraction of the glycerol supply is used for pyruvate formation as a precursor in DAP biosynthesis which is also required for the peptidoglycan layer of *C. burnetii*. Interestingly, the amount of peptidoglycan in the SCV of this pathogen is about 2.7-fold higher than the amount in the LCV (Amano et al., 1984). This could indicate that glycerol becomes a more important substrate at later stages during the developmental cycle of *C. burnetii*, e.g., when the formation of the SCV is initialized. Like *L. pneumophila*, also *C. burnetii* uses Ser for energy generation in the TCA cycle (Eylert et al., 2010; Häuslein et al., 2015; Gillmaier et al., 2016). In contrast to *L. pneumophila*, however, almost no carbon flux occurred from Ser into gluconeogenetic reactions, indicating that the metabolism of Ser is more restricted to energy generation in *C. burnetii* than in *L. pneumophila*.

**Figure 5:**
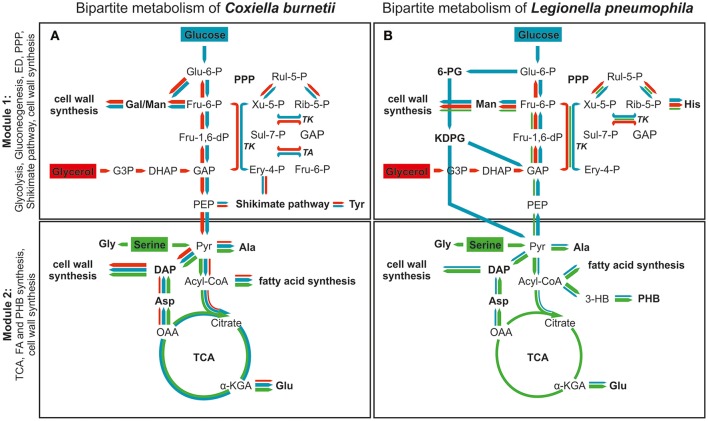
The bipartite metabolic model of *C. burnetii* **(A)** and *L. pneumophila* **(B)**. The network is divided into two modules. Module 1 comprises glycolytic and gluconeogenetic reactions, the ED pathway, the shikimate/chorismate pathway as well as the biosynthesis of cell wall components. Module 2 includes the TCA cycle, fatty acid and PHB biosynthesis as well as the biosynthesis of the central cell wall component DAP. Color and size of arrows indicate direction and intensity of carbon flux from serine (green), glucose (blue) and glycerol (red). Serine serves as a main carbon and energy source which is predominantly metabolized in the TCA cycle for energy generation in both pathogens (Module 2). Nevertheless, in *C. burnetii* serine is not metabolized in as high rates as in *L. pneumophila*. Furthermore, carbon flux from this amino acid occurs exclusively into the second part of metabolism (TCA cycle) in *C. burnetii*, whereas carbon flux from serine also occurs into the upper part of metabolism in *L. pneumophila*. Glucose catabolism differs in both pathogenic bacteria. While *C. burnetii* degrades glucose in glycolytic reactions, *L. pneumophila* uses predominantly the Entner-Doudoroff pathway. Thereby, *C. burnetii* uses glucose very efficiently in both parts of metabolism, also for tyrosine biosynthesis via the shikimate pathway. In contrast, usage of glucose by *L. pneumophila* is much lower and more restricted toward the upper part of metabolism. Glycerol is metabolized at very low rates by *L. pneumophila* and thereby used exclusively in gluconeogenetic reactions and the PPP. In contrast, it effectively serves as carbon source for both modules in *C. burnetii*, and also for tyrosine biosynthesis in the shikimate pathway. Furthermore, glycerol is preferably used for cell wall biosynthesis by *C. burnetii*. α-KGA, α-ketoglutarate; DAP, diaminopimelate; Pyr, pyruvate; OAA, oxaloacetate; Acyl-CoA, acetyl-CoA, Man, mannose; Gal, galactose; 3-HB, 3-hydroxybutyrate; PHB, poly-hydroxybutyrate; PEP, phosphoenolpyruvate; GAP, glyceraldehyde 3-phosphate; DHAP, dihydroxyacetone phosphate; TK, transketolase; TA, transaldolase; G3P, glycerol 3-phosphate; PPP, pentose phosphate pathway; TCA, TCA cycle.

Altogether, *C. burnetii* features a bipartite-type metabolic network which is similar but not identical to that reported recently for its close relative *L. pneumophila* (Schunder et al., 2014; Häuslein et al., 2015) (Figure 5). Section 1 of the partite model comprises glycolytic and gluconeogenetic reactions, cell wall biosynthesis derived from hexoses, the ED pathway (in the case of *L. pneumophila*), the PPP and the shikimate pathway (in the case of *C. burnetii*). On the other hand, section 2 includes the TCA cycle, fatty acid and amino acid biosynthesis using acetyl-CoA or TCA cycle derived intermediates as precursors. On this basis, section 2 of the model represents more the energy generating part, since high amounts of ATP, NADH/H^+^ and FADH_2_ are derived from the TCA cycle and the electron transfer chain. In section 1, however, high amounts of NADPH/H^+^ are produced via the PPP, and this section predominantly serves anabolic purposes and more represents the energy consuming part of metabolism.

In *C. burnetii* and *L. pneumophila*, glycerol and glucose are preferably shuffled into section 1 of the metabolic network predominantly serving anabolic processes, especially cell wall biosynthesis. Serine on the other hand is used in section 2 of the network with the TCA cycle for energy generation (Eylert et al., 2010; Häuslein et al., 2015; Gillmaier et al., 2016) (Figure 5).

Notably and despite the fact that *C. burnetii* and *L. pneumophila* are close relatives, there are also considerable differences in their metabolic concepts, since glucose and glycerol are more important nutrients for *C. burnetii* than for *L. pneumophila*. Specifically, in *C. burnetii*, glucose can also be shuffled efficiently into section 2 of the metabolism while in *L. pneumophila* this section is quite restricted for the usage of serine as carbon supply (Keen and Hoffman, 1984).

In *L. pneumophila*, all enzymes for glycolytic reactions as well as for the ED pathway are reflected in their genomes (Cazalet et al., 2004; Chien et al., 2004), whereas in *C. burnetii* genes encoding the ED pathway are partly missing (Seshadri et al., 2003). However, in a striking difference, glucose catabolism occurs via the ED pathway in *L. pneumophila* and via glycolysis in *C. burnetii* (McDonald and Mallavia, 1971; Eylert et al., 2010) (Figure 5).

The directed carbon flux from the carbon substrates under study furthermore suggests a growth phase dependent utilization of these nutrients under intracellular conditions. Since Ser is predominantly shuffled into the TCA cycle via pyruvate and acetyl-CoA for e.g., energy generation via NADH and respiration, these metabolic processes are predominantly induced during early replication in the CCV, when *C. burnetii* appears in its LCV (Coleman et al., 2007). In contrast, glucose and glycerol could preferably be used at later growth phases than serine, probably when the cells start to develop into the SCV, since these precursors predominantly serve the biosynthesis of peptidoglycan, which is present in higher amounts in this morphological form (Amano et al., 1984).

Interestingly, enzymes for the biosynthesis of the energy storage compound poly-3-hydroxybutyrate (PHB) are not present in *C. burnetii* (Seshadri et al., 2003), whereas its biosynthesis plays a central role in the metabolism and life cycle of *L. pneumophila* (Keen and Hoffman, 1984; Gillmaier et al., 2016). Furthermore, degradation of PHB by *L. pneumophila* seems to be an important factor for long term survival of this intracellular pathogen in the environment (Li et al., 2015). For biosynthesis of this storage compound, which is predominantly produced during the late growth phase, *L. pneumophila* preferably uses serine and glucose to provide acetyl-CoA as the building units for 3-hydroxybutyrate and its polymer (Eisenreich and Heuner, 2016; Gillmaier et al., 2016). The reason for the apparent inability of *C. burnetii* to build PHB is unknown, but could be linked to the acidified conditions in the CCV. Nevertheless, this raises questions about the long-term survival strategies of *C. burnetii* (Seshadri et al., 2003).

A further difference in the metabolic potential of these two intracellular pathogens is the active shikimate pathway in *C. burnetii*. Our data clearly show that *C. burnetii* is able to synthesize Tyr *de novo* via this metabolic pathway and is thus not auxotroph for Tyr as it was concluded from recent growth experiments in the minimal defined medium ACCM-D (Sandoz et al., 2016a). In contrast, *L. pneumophila* seems to be auxotroph for the biosynthesis of aromatic amino acids (Eylert et al., 2010; Häuslein et al., 2015; Gillmaier et al., 2016) at least under *in vitro* conditions, although almost all enzymes of this biosynthetic route appear to be present based on the genome sequence (Chien et al., 2004). However, the metabolic role or importance of the shikimate pathway in *C. burnetii* or *L. pneumophila* has not been studied extensively until now. On one hand, *in vitro* grown *L. pneumophila* does not need to use this pathway for the *de novo* biosynthesis of aromatic amino acids and rather imports these amino acids from the medium. On the other hand, recent studies demonstrated that mutants of *L. pneumophila* concerning two enzymes of the shikimate pathway, *aroB* and *aroE*, were defective in infection and replication inside of human macrophages (Jones et al., 2015).

Since the shikimate pathway is not present in mammalian, this biosynthetic pathway is a potential target for drug development agains pathogenic bacteria (Consigli and Paretsky, 1962). Especially the enolpyruvylshikimate 3-phosphate synthase (EPSP synthase) that catalyzes the biosynthesis of 5-enolpyruvylshikimate 3-phosphate from shikimate 3-phosphate and PEP, which is subsequently converted to chorismate in the downstream reactions of this metabolic pathway, could be a promising target for new antibiotics against *C. burnetii* and further pathogens (Ferreras et al., 2005; Lemaître et al., 2014; Light et al., 2016). In general, the EPSP synthases are divided into two classes dependent on their sensitivity toward the herbicide glyphosate. Thereby, class I EPSP synthases, which are present in plants and some Gram positive bacteria, are highly sensitive toward this herbicide, whereas class II EPSP synthases retain their catalytic activity in the presence of glyphosate (Franklin et al., 2015; Light et al., 2016). These class II EPSP synthases, which have been found in some glyphosate-tolerant bacteria, also differ in their genome sequence compared to the class I enzymes (Duke and Powles, 2008; Pollegioni et al., 2011). However, class II EPSP synthases, like the synthase of *C. burnetii* (CBU0526) are not as well studied as the well-known class I EPSP synthases (Duke and Powles, 2008; Pollegioni et al., 2011).

Nevertheless, besides the EPSP synthases also other enzymes of this biosynthetic pathway represent potential drug targets in various pathogens including *Mycobacterium tuberculosis* and *Helicobacter pylori* and are in the focus of current research (Parish and Stoker, 2002; Ducati et al., 2007; Vianna and De Azevedo, 2012; Blanco et al., 2013). Notably, mutations in the gene encoding the type-II EPSP synthase (CBU0526) and the shikimate dehydrogenase of *C. burnetii* (CBU0010) also showed significant intracellular growth defects (Martinez et al., 2014, and unpublished results), strongly suggesting that the shikimate route is indeed essential for intracellular *C. burnetii*.

In summary, *C. burnetii* features a bipartite metabolic network resembling the topology of its closely relative *L. pneumophila* with amino acids as major nutrients. However, in comparison to *L. pneumophila, C. burnetii* shows higher rates in the usages of glucose and glycerol, especially for cell wall biosynthesis and for the synthesis of tyrosine. These differences could reflect the lower replication rates of *C. burnetii* in comparison to *L. pneumophila*, thereby imprinting a lower burden to adapt to the limited nutrient supply under intracellular conditions. However, the general concept of multi-substrate usage in a bipartite metabolic network is also valid for *C. burnetii* and could benefit the robustness and survival of the intracellular pathogen.

## Author contributions

WE, IH, FrC, and MB designed the study and wrote the manuscript. IH, FrC, SR, and FaC performed the experimental work.

### Conflict of interest statement

The authors declare that the research was conducted in the absence of any commercial or financial relationships that could be construed as a potential conflict of interest.
