# Supplementary material for: Multiple Substrate Usage of Coxiella burnetii to Feed a Bipartite Metabolic Network
Source: Front Cell Infect Microbiol. 2017 Jun 29;7:285. doi: 10.3389/fcimb.2017.00285 (PMC5489692; doi:10.3389/fcimb.2017.00285)
Supplement: Supplementary file 1 [file DataSheet1.PDF]

Supplementary Material

Multiple substrate usage of *Coxiella burnetii* to feed a bipartite metabolic network

Ina Häuslein [1], Franck Cantet[2], Sarah Reschke[1], Fan Chen[1], Matteo Bonazzi[2*], and Wolfgang Eisenreich[1*]

*** Correspondence:**
Wolfgang Eisenreich
wolfgang.eisenreich@ch.tum.de
Matteo Bonazzi
Matteo.bonazzi@cpbs.cnrs.fr

Supplementary Figures and Tables

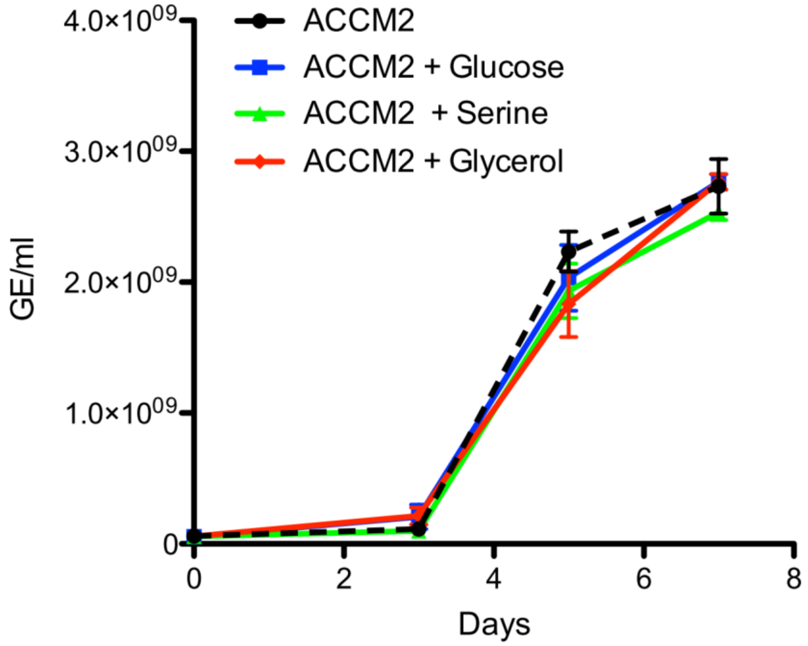

Supplementary Figure 1: Growth curves of *C. burnetii* in ACCM-2 in the presence of additional 5 mM glucose, serine or glycerol. To analyze the effect of additional amounts of serine, glucose or glycerol on the *in vitro* growth of *C. burnetii*, Acidified Citrate Cysteine Medium 2 (ACCM-2, **Table S1**) was supplemented with each substrate at 5 mM and inoculated with *Coxiella burnetii* RSA 493 NMII at $2x10^7$ GE/mL. Bacteria were cultured in a humidified atmosphere of 5% CO_2 and 2.5% O_2 at 37°C and replication was assessed at 0, 3, 5, 7 days post-inoculation using the PicoGreen assay as previously described (Martinez et al., 2014). Values are means ± SD of three independent experiments.

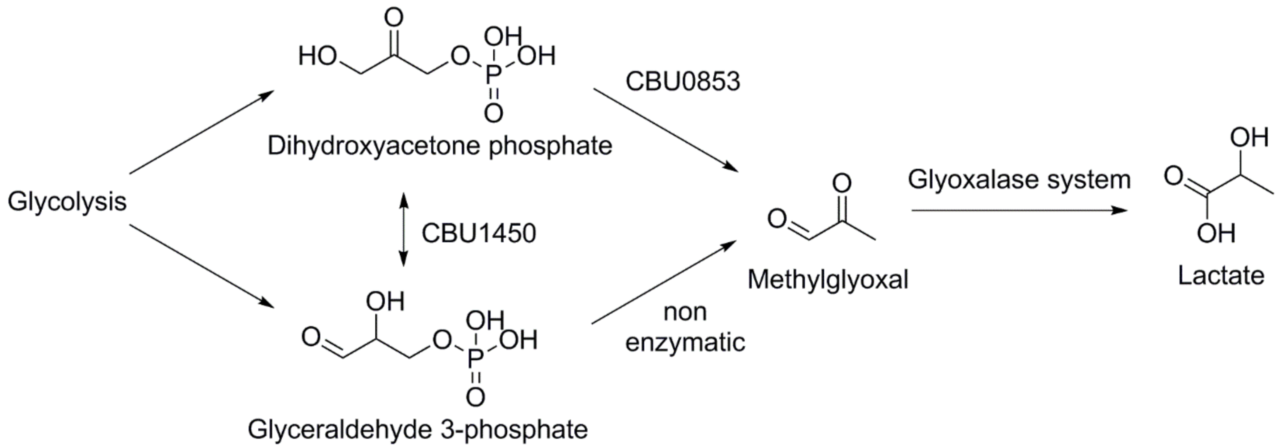

Supplementary Figure S2: Proposed metabolism of methylglyoxal/lactate by *C. burnetii* (Wattam et al., 2013; Kanehisa et al., 2017). Lactate is probably built *via* methylglyoxal in *C. burnetii*, since enzymes for the direct synthesis from pyruvate have not been annotated yet in this pathogen. Methylglyoxal is a known byproduct of metabolic processes and can be generally built non enzymatically from intermediates of glycolysis (dihydroxyacetone phosphate and glyceraldehyde 3-phosphate). Furthermore, *C. burnetii* features a methylglyoxal synthase (CBU0853), which could catalyze the formation of methylglyoxal from dihydroxyacetone phosphate. Since methylglyoxal is toxic, it is typically degraded in a detoxification process *via* the glyoxalase system. However, one enzyme (glyoxalase I) seems to be missing, whereas the second enzyme (glyoxalase II, CBU0314) is annotated in *C. burnetii*.

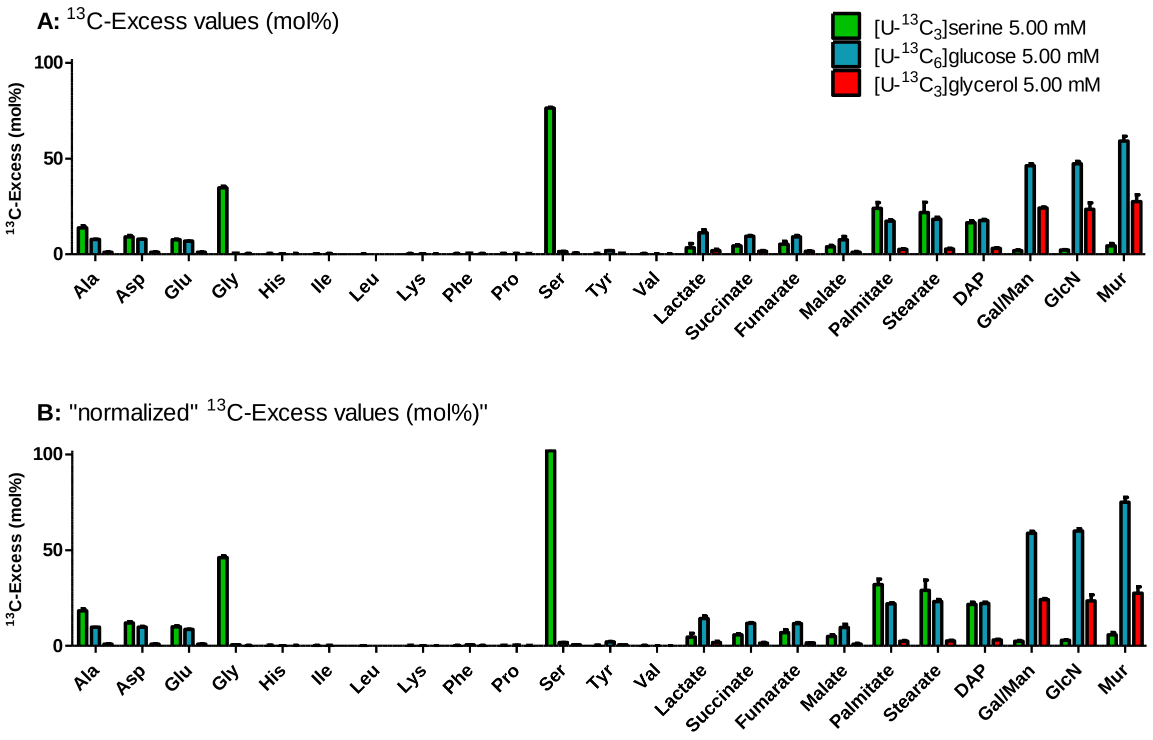

Supplementary Figure S3: Comparison of ^{13}C-excess values (mol%) from labeling experiments to "normalized" values (mol%). (**A**) ^{13}C-Excess values (mol%) of protein-derived amino acids, methanol-soluble polar metabolites, DAP, Gal/Man, GlcN and Mur from experiments with *Coxiella burnetii* RSA 493 NMII wild-type grown in ACCM-2 supplemented with 5 mM [U-$^{13}C_3$]serine, 5 mM [U-$^{13}C_6$]glucose or 5 mM [U-$^{13}C_3$]glycerol. (**B**) To estimate the carbon fluxes into these key metabolites under the experimental conditions, the ^{13}C-excess values (mol%) from the experiments were multiplied ("normalized") with the respective factors (^{13}C-serine: 1.336; ^{13}C-glucose: 1.270) to calculate the values (mol%) that would be reached if only labeled free serine or glucose would be present in the ACCM-2 medium. ACCM-2 comprises 1.68 mM free serine and 1.39 mM free glucose (Sales et al., 1995; Omsland et al., 2011; Sandoz et al., 2016). Since no glycerol is present in this medium, this calculation is not needed for the experiment with ^{13}C-glycerol. *Abbreviations*: DAP, diaminopimelic acid; Man, mannose; Gal, galactose; GlcN, glucosamine; Mur, muramic acid.

Table S1: Composition of Acidified Citrate Cysteine Medium 2 (ACCM-2). All components are dissolved in 1000 mL ddH$_2$0. The pH is adjusted to 4.75 and the solution is sterilized by filtration.

Component	[mg/L]	[mol/L]
Citric acid	2568.00	0.0134000
Sodium citrate	4740.00	0.0161000
Potassium phosphate	500.00	0.0036700
Magnesium chloride	200.00	0.0009800
Calcium chloride	13.20	0.0000197
Iron sulfate	2.78	0.0000100
Sodium chloride	7280.00	0.1254000
L-cysteine	263.40	0.0015000
Bacto Neopeptone	100.00	n/a
Casamino acids	2500.00	n/a
Methyl-B-Cyclodextrin	1000.00	
RPMI w/glutamax	125.00	n/a
Deionized H_2O	865.00	n/a

Table S2: Retention times in the GC-MS runs and mass fragments of TBDMS-derivatives of amino acids and methanol soluble polar metabolites and TMS-derivatives of cell wall derived hexoses (Man/Gal, GlcN and Mur) used for isotopologue calculations.

Metabolite	RT [min]	$[M-15]^+$	$[M-57]^+$	$[M-85]^+$	$[M-176]^+$
**Ala**	6.7		m/z 260		
**Gly**	7.0		m/z 246		
**Val**	8.5		m/z 288		
**Leu**	9.1			m/z 274	
**Ile**	9.5			m/z 274	
**Pro**	10.1		m/z 286		
**Ser**	13.2		m/z 390		
**Phe**	14.5		m/z 336		
**Asp**	15.4		m/z 418		
**Glu**	16.8		m/z 432		
**Lys**	18.1		m/z 431		
**His**	20.4		m/z 440		
**Tyr**	21.0		m/z 466		
**DAP**	23.4		m/z 589		
**Lactate**	17.8		m/z 261		
**Succinate**	27.5		m/z 289		
**Fumarate**	28.7		m/z 287		
**Malate**	39.1		m/z 419		
**Palmitate**	44.0		m/z 313		
**Stearate**	49.4		m/z 341		
**Man/Gal**	8.7	m/z 287			
**GlcN**	32.6	m/z 452			
**Mur**	36.7				m/z 434

Table S3: **^{13}C-Excess (mol%) of protein-derived amino acids, methanol-soluble polar metabolites, diaminopimelate (DAP), mannose/galactose (Man/Gal), glucosamine (GlcN) and muramic acid (Mur) from experiments with *Coxiella burnetii* RSA 493 NMII wild-type grown in ACCM-2 supplemented with 5 mM [U-$^{13}C_3$]serine, 5 mM [U-$^{13}C_6$]glucose or 5 mM [U-$^{13}C_3$]glycerol.** Cells were harvested after 7 days of growth at 37°C. Data are means and standard deviations of six values (3 technical replicates x 2 biological replicates).

^{13}C-Excess (mol%)	**[U-$^{13}C_3$]serine**	**[U-$^{13}C_6$]glucose**	**[U-$^{13}C_3$]glycerol**
**Ala**	13.78% ± 1.02%	7.73% ± 0.11%	1.04% ± 0.12%
**Asp**	8.99% ± 0.70%	7.79% ± 0.17%	0.99% ± 0.16%
**Glu**	7.47% ± 0.48%	6.83% ± 0.08%	1.02% ± 0.10%
**Gly**	34.59% ± 0.86%	0.48% ± 0.10%	0.16% ± 0.07%
**His**	0.23% ± 0.19%	0.11% ± 0.05%	0.10% ± 0.14%
**Ile**	0.12% ± 0.08%	0.19% ± 0.05%	0.05% ± 0.02%
**Leu**	0.09% ± 0.09%	0.03% ± 0.02%	0.01% ± 0.01%
**Lys**	0.18% ± 0.11%	0.11% ± 0.03%	0.10% ± 0.03%
**Phe**	0.16% ± 0.10%	0.48% ± 0.11%	0.19% ± 0.04%
**Pro**	0.25% ± 0.06%	0.37% ± 0.05%	0.24% ± 0.02%
**Ser**	76.35% ± 0.31%	1.37% ± 0.08%	0.64% ± 0.11%
**Tyr**	0.26% ± 0.14%	1.75% ± 0.09%	0.58% ± 0.02%
**Val**	0.15% ± 0.15%	0.08% ± 0.03%	0.08% ± 0.04%
**Lactate**	3.41% ± 2.11%	11.27% ± 1.44%	1.87% ± 0.63%
**Succinate**	4.37% ± 0.46%	9.35% ± 0.44%	1.50% ± 0.30%
**Fumarate**	5.25% ± 1.51%	9.16% ± 0.64%	1.61% ± 0.02%
**Malate**	3.79% ± 0.74%	7.62% ± 1.64%	1.01% ± 0.63%
**Palmitate**	23.98% ± 2.95%	17.31% ± 0.62%	2.58% ± 0.23%
**Stearate**	21.77% ± 5.28%	18.27% ± 1.01%	2.60% ± 0.41%%
**DAP**	16.32% ± 1.18%	17.51% ± 0.60%	3.13% ± 0.30%
**Man/Gal**	1.84% ± 0.34%	46.34% ± 0.92%	24.14% ± 0.59%
**GlcN**	2.25% ± 0.14%	47.22% ± 1.26%	23.47% ± 3.27%
**Mur**	4.37% ± 1.15%	59.09% ± 2.56%	27.53% ± 3.45%

Table S4: **Normalized ^{13}C-incorporation (mol%) of protein-derived amino acids, methanol-soluble polar metabolites, diaminopimelate (DAP), mannose/galactose (Man/Gal), glucosamine (GlcN) and muramic acid (Mur) from experiments with *Coxiella burnetii* RSA 493 NMII wild-type grown in ACCM-2 supplemented with 5 mM [U-$^{13}C_3$]serine, 5 mM [U-$^{13}C_6$]glucose or 5 mM [U-$^{13}C_3$]glycerol.** To evaluate the incorporation (%) into these key metabolites under the experimental conditions, the ^{13}C-excess values (mol%) from the experiments were multiplied with the respective factor (^{13}C-serine: 1.336; ^{13}C-glucose: 1.270) to calculate the ^{13}C-excess values (mol%) that would be reached if only labeled free serine or glucose would be present in the axenic media. ACCM-2 comprises 1.68 mM free serine and 1.39 mM free glucose. Since no glycerol is present in this media, this calculation is not needed for the experiment with ^{13}C-glycerol.

^{13}C-Excess (mol%)	**[U-$^{13}C_3$]serine**	**[U-$^{13}C_6$]glucose**	**[U-$^{13}C_3$]glycerol**
**Ala**	18.41% ± 1.02%	9.82% ± 0.11%	1.04% ± 0.12%
**Asp**	12.01% ± 0.70%	9.89% ± 0.17%	0.99% ± 0.16%
**Glu**	9.98% ± 0.48%	8.68% ± 0.08%	1.02% ± 0.10%
**Gly**	46.22% ± 0.86%	0.60% ± 0.10%	0.16% ± 0.07%
**His**	0.30% ± 0.19%	0.14% ± 0.05%	0.10% ± 0.14%
**Ile**	0.16% ± 0.08%	0.24% ± 0.05%	0.05% ± 0.02%
**Leu**	0.12% ± 0.09%	0.04% ± 0.02%	0.01% ± 0.01%
**Lys**	0.24% ± 0.11%	0.14% ± 0.03%	0.10% ± 0.03%
**Phe**	0.21% ± 0.10%	0.61% ± 0.11%	0.19% ± 0.04%
**Pro**	0.34% ± 0.06%	0.47% ± 0.05%	0.24% ± 0.02%
**Ser**	102.00% ± 0.31%	1.74% ± 0.08%	0.64% ± 0.11%
**Tyr**	0.35% ± 0.14%	2.22% ± 0.09%	0.58% ± 0.02%
**Val**	0.20% ± 0.15%	0.10% ± 0.03%	0.08% ± 0.04%
**Lactate**	4.55% ± 2.11%	14.32% ± 1.44%	1.87% ± 0.63%
**Succinate**	5.84% ± 0.46%	11.87% ± 0.44%	1.50% ± 0.30%
**Fumarate**	7.02% ± 1.51%	11.64% ± 0.64%	1.61% ± 0.02%
**Malate**	5.06% ± 0.74%	9.68% ± 1.64%	1.01% ± 0.63%
**Palmitate**	32.03% ± 2.95%	21.99% ± 0.62%	2.58% ± 0.23%
**Stearate**	29.09% ± 5.28%	23.20% ± 1.01%	2.60% ± 0.41%%
**DAP**	21.80% ± 1.18%	22.24% ± 0.06%	3.13% ± 0.30%
**Man/Gal**	2.46% ± 0.34%	58.86% ± 0.92%	24.14% ± 0.59%
**GlcN**	3.00% ± 0.14%	59.97% ± 1.26%	23.47% ± 3.27%
**Mur**	5.84% ± 1.15%	75.05% ± 2.56%	27.53% ± 3.45%

Table S5: Relative fractions of isotopologues (mol%) in protein-derived amino acids, methanol-soluble polar metabolites, diaminopimelate (DAP), mannose/galactose (Man/Gal), glucosamine (GlcN) and muramic acid (Mur) from experiments with *Coxiella burnetii* RSA 493 NMII wild-type grown in ACCM-2 supplemented with 5 mM [U-$^{13}C_3$]serine. M+X represents the mass of the unlabeled metabolite plus X labeled ^{13}C-atoms. Shown are mean and standard deviations of six values (3 technical replicates x 2 biological replicates

5 mM [U-$^{13}C_3$]serine										
	**Ala**	**Asp**	**Glu**	**Gly**	**Ser**	**Tyr**	**Lactate**	**Succinate**	**Fumarate**	**Malate**
**M+1**	2.26% ± 0.28%	8.64% ± 0.51%	5.71% ± 0.44%	3.05% ± 0.33%	1.73% ± 0.14%		0.02% ± 0.05%	4.40% ± 1.19%	3.36% ± 0.71%	3.78% ± 1.38%
**M+2**	1.50% ± 0.13%	10.21% ± 0.74%	11.11% ± 0.61%	33.07% ± 0.70%	3.12% ± 0.09%		0.28% ± 0.22%	5.20% ± 0.50%	6.71% ± 2.15%	3.95% ± 1.05%
**M+3**	12.03% ± 0.90%	2.09% ± 0.23%	1.81% ± 0.18%		73.69% ± 0.42%		3.21% ± 1.97%	0.87% ± 0.22%	1.36% ± 0.37%	0.97% ± 0.31%
**M+4**		0.16% ± 0.06%	0.87% ± 0.18%					0.02% ± 0.02%	0.03% ± 0.07%	0.14% ± 0.10%
**M+5**			0.10% ± 0.01%							
**M+6**										
**M+7**										
**M+8**										
**M+9**										
	**Palmitate**	**Stearate**	**DAP**	**Man/Gal**	**GlcN**	**Mur**				
**M+1**	2.74% ± 0.71%	2.63% ± 0.58%	9.09% ± 0.71%							
**M+2**	11.73% ± 0.94%	8.87% ± 1.62%	9.64% ± 0.30%							
**M+3**	7.28% ± 0.88%	6.20% ± 1.48%	19.51% ± 1.44%							
**M+4**	14.95% ± 1.58%	12.51% ± 2.77%	2.59% ± 0.53%							
**M+5**	8.29% ± 1.06%	7.87% ± 1.95%	2.69% ± 0.31%							
**M+6**	12.24% ± 1.56%	11.23% ± 2.75%	0.51% ± 0.04%							
**M+7**	5.75% ± 0.75%	6.20% ± 1.56%	0.06% ± 0.03%							
**M+8**	6.66% ± 0.90%	7.06% ± 1.76%								
**M+9**	2.57% ± 0.38%	3.36% ± 0.87%								
**M+10**	2.51% ± 0.36%	3.16% ± 0.82%								
**M+11**	0.74% ± 0.10%	1.24% ± 0.34%								
**M+12**	0.60% ± 0.08%	1.00% ± 0.27%								
**M+13**	0.12% ± 0.02%	0.32% ± 0.09%								
**M+14**	0.07% ± 0.02%	0.21% ± 0.06%								
**M+15**	0.02% ± 0.01%	0.05% ± 0.02%								
**M+16**	0.07% ± 0.08%	0.04% ± 0.01%								
**M+17**		0.01% ± 0.01%								
**M+18**		0.01% ± 0.02%								

Table S6: Relative fractions of isotopologues (mol%) of protein-derived amino acids, methanol-soluble polar metabolites, diaminopimelate (DAP), mannose/galactose (Man/Gal), glucosamine (GlcN) and muramic acid (Mur) from experiments with *Coxiella burnetii* RSA 493 NMII wild-type grown in ACCM-2 supplemented with 5 mM [U-$^{13}C_6$]glucose. M+X represents the mass of the unlabeled metabolite plus X labeled ^{13}C-atoms. Shown are mean and standard deviations of six values (3 technical replicates x 2 biological replicates.

5 mM [U-$^{13}C_6$]glucose										
	**Ala**	**Asp**	**Glu**	**Gly**	**Ser**	**Tyr**	**Lactate**	**Succinate**	**Fumarate**	**Malate**
**M+1**	1.42% ± 0.19%	7.74% ± 0.17%	5.28% ± 0.42%	0.34% ± 0.10%	0.01% ± 0.03%	0.31% ± 0.27%	0.36% ± 0.16%	5.77% ± 0.19%	5.85% ± 0.32%	5.51% ± 2.05%
**M+2**	1.06% ± 0.07%	8.74% ± 0.11%	10.19% ± 0.14%	0.31% ± 0.06%	0.00% ± 0.00%	0.54% ± 0.15%	0.97% ± 0.23%	9.95% ± 0.62%	9.22% ± 0.77%	7.63% ± 1.50%
**M+3**	6.55% ± 0.04%	1.86% ± 0.04%	1.62% ± 0.12%		1.36% ± 0.08%	0.91% ± 0.10%	10.51% ± 1.27%	3.53% ± 0.13%	3.49% ± 0.29%	2.66% ± 0.48%
**M+4**		0.09% ± 0.10%	0.77% ± 0.03%			0.67% ± 0.04%		0.28% ± 0.04%	0.47% ± 0.04%	0.44% ± 0.11%
**M+5**			0.12% ± 0.02%			0.65% ± 0.05%				
**M+6**						0.56% ± 0.04%				
**M+7**						0.17% ± 0.05%				
**M+8**						0.14% ± 0.04%				
**M+9**						0.00% ± 0.00%				

	**Palmitate**	**Stearate**	**DAP**	**Man/Gal**	**GlcN**	**Mur**
**M+1**	2.47% ± 0.19%	2.96% ± 0.09%	9.04% ± 1.01%	10.75% ± 0.65%	12.16% ± 0.41%	5.71% ± 2.14%
**M+2**	13.92% ± 1.24%	11.79% ± 0.45%	9.62% ± 0.48%	11.70% ± 0.37%	13.52% ± 0.21%	12.29% ± 1.81%
**M+3**	6.18% ± 0.21%	6.37% ± 0.13%	20.27% ± 0.57%	26.77% ± 0.61%	22.79% ± 1.82%	19.37% ± 2.83%
**M+4**	13.42% ± 0.73%	13.01% ± 0.25%	3.12% ± 0.43%	11.71% ± 0.36%	11.40% ± 0.47%	23.03% ± 6.27%
**M+5**	5.74% ± 0.13%	6.67% ± 0.22%	2.95% ± 0.33%	9.66% ± 0.52%	14.31% ± 2.02%	10.78% ± 1.32%
**M+6**	8.67% ± 0.24%	9.66% ± 0.28%	0.87% ± 0.22%	11.41% ± 0.49%	9.77% ± 0.66%	20.03% ± 1.57%
**M+7**	3.42% ± 0.37%	4.61% ± 033%	0.15% ± 0.08%			
**M+8**	4.03% ± 0.41%	5.23% ± 0.40%				
**M+9**	1.43% ± 0.26%	2.26% ± 0.30%				
**M+10**	1.41% ± 0.23%	2.15% ± 0.30%				
**M+11**	0.43% ± 0.12%	0.83% ± 0.18%				
**M+12**	0.35% ± 0.11%%	0.69% ± 0.17%				
**M+13**	0.08% ± 0.03%	0.23% ± 0.07%				
**M+14**	0.04% ± 0.02%	0.16% ± 0.05%				
**M+15**	0.00% ± 0.01%	0.05% ± 0.01%				
**M+16**	0.00% ± 0.00%	0.03% ± 0.01%				
**M+17**		0.01% ± 0.00%				
**M+18**		0.00% ± 0.00%				

Table S7: Relative fractions of isotopologues (mol%) of protein-derived amino acids, methanol-soluble polar metabolites, diaminopimelate (DAP), mannose/galactose (Man/Gal), glucosamine (GlcN) and muramic acid (Mur) from experiments with *Coxiella burnetii* RSA 493 NMII wild-type grown in ACCM-2 supplemented with 5 mM [U-$^{13}C_3$]glycerol. M+X represents the mass of the unlabeled metabolite plus X labeled ^{13}C-atoms. Shown are mean and standard deviations of six values (3 technical replicates x 2 biological replicates.

5 mM [U-$^{13}C_3$]glycerol										
	**Ala**	**Asp**	**Glu**	**Gly**	**Ser**	**Tyr**	**Lactate**	**Succinate**	**Fumarate**	**Malate**
**M+1**	0.62% ± 0.38%	1.95% ± 0.45%	1.15% ± 0.47%		0.00% ± 0.00%	0.69% ± 0.24%	0.11% ± 0.19%	0.45% ± 0.74%	0.12% ± 0.20%	
**M+2**	0.16% ± 0.05%	0.84% ± 0.13%	1.80% ± 0.18%		0.00% ± 0.00%	0.48% ± 0.19%	0.64% ± 0.29%	1.57% ± 0.38%	1.81% ± 0.21%	
**M+3**	0.73% ± 0.04%	0.11% ± 0.04%	0.06% ± 0.05%		0.64% ± 0.11%	0.46% ± 0.08%	1.40% ± 0.43%	0.73% ± 0.32%	0.83% ± 0.05%	
**M+4**		0.00% ± 0.00%	0.00% ± 0.00%			0.17% ± 0.05%		0.06% ± 0.08%	0.05% ± 0.07%	
**M+5**			0.03% ± 0.03%			0.21% ± 0.04%				
**M+6**						0.05% ± 0.03%				
**M+7**						0.00% ± 0.00%				
**M+8**						0.01% ± 0.02%				
**M+9**						0.01% ± 0.01%				
	**Palmitate**	**Stearate**	**DAP**	**Man/Gal**	**GlcN**	**Mur**				
**M+1**	3.34% ± 0.68%	5.45% ± 1.26%	3.50% ± 1.35%	16.25% ± 0.64%	15.92% ± 0.33%	15.02% ± 4.69%				
**M+2**	13.08% ± 1.41%	13.28% ± 2.22%	3.10% ± 0.16%	13.48% ± 0.48%	12.77% ± 0.51%	14.89% ± 1.26%				
**M+3**	1.13% ± 0.07%	1.55% ± 0.25%	3.66% ± 0.36%	20.37% ± 1.15%	18.28% ± 0.93%	14.34% ± 5.79%				
**M+4**	1.63% ± 0.10%	1.84% ± 0.25%	0.20% ± 0.11%	3.90% ± 0.30%	3.53% ± 0.35%	10.42% ± 2.32%				
**M+5**	0.17% ± 0.01%	0.24% ± 0.03%	0.06% ± 0.06%	2.42% ± 0.25%	2.28% ± 0.21%	2.55% ± 1.96%				
**M+6**	0.13% ± 0.01%	0.20% ± 0.02%	0.01% ± 0.02%	2.13% ± 0.31%	1.82% ± 0.25%	3.83% ± 1.49%				
**M+7**	0.01% ± 0.01%	0.02% ± 0.00%	0.00% ± 0.00%							
**M+8**	0.00% ± 0.00%	0.02% ± 0.00%								
**M+9**	0.00% ± 0.00%	0.00% ± 0.00%								
**M+10**	0.00% ± 0.00%	0.00% ± 0.00%								
**M+11**	0.00% ± 0.00%	0.00% ± 0.00%								
**M+12**	0.00% ± 0.00%	0.00% ± 0.00%								
**M+13**	0.00% ± 0.00%	0.00% ± 0.00%								
**M+14**	0.00% ± 0.00%	0.00% ± 0.00%								
**M+15**	0.00% ± 0.00%	0.00% ± 0.00%								
**M+16**	0.00% ± 0.01%	0.00% ± 0.00%								
**M+17**		0.00% ± 0.00%								
**M+18**		0.00% ± 0.00%								

Table S8: Ratio of ^{13}C-excess in diaminopimelate (DAP) to ^{13}C-excess in Ala calculated from experiments with *Coxiella burnetii* RSA 493 NMII wild-type grown in ACCM-2 supplemented with 5 mM [U-$^{13}C_3$]serine, 5 mM [U-$^{13}C_6$]glucose or 5 mM [U-$^{13}C_3$]glycerol. Cells were harvested after 7 days of growth at 37°C. Standard deviation was calculated from the highest possible (+) and the lowest possible (-) value.

**Ratio**: ^{13}C-excess (mol%) DAP/^{13}C-excess (mol %) Ala								
**5 mM [U-$^{13}C_3$]serine**	**+**	**-**	**5 mM [U-$^{13}C_6$]glucose**	**+**	**-**	**5 mM [U-$^{13}C_3$]glycerol**	**+**	**-**
1.18	0.19	0.16	2.27	0.11	0.11	3.01	0.72	0.57

Table S9: Ratio of ^{13}C-excess in glucosamine (GlcN) to ^{13}C-excess in Ala calculated from experiments with *Coxiella burnetii* RSA 493 NMII wild-type grown in ACCM-2 supplemented with 5 mM [U-$^{13}C_3$]serine, 5 mM [U-$^{13}C_6$]glucose or 5 mM [U-$^{13}C_3$]glycerol. Cells were harvested after 7 days of growth at 37°C. Standard deviation was calculated from the highest possible (+) and the lowest possible (-) value.

**Ratio**: ^{13}C-excess (mol%) GlcN/^{13}C-excess (mol %) Ala								
**5 mM [U-$^{13}C_3$]serine**	**+**	**-**	**5 mM [U-$^{13}C_6$]glucose**	**+**	**-**	**5 mM [U-$^{13}C_3$]glycerol**	**+**	**-**
0.16	0.02	0.02	6.11	0.25	0.25	22.61	6.51	5.16

Kanehisa, M., Furumichi, M., Tanabe, M., Sato, Y., and Morishima, K. (2017). KEGG: new perspectives on genomes, pathways, diseases and drugs. *Nucleic Acids Research* 45**,** D353-D361.

Omsland, A., Beare, P.A., Hill, J., Cockrell, D.C., Howe, D., Hansen, B., Samuel, J.E., and Heinzen, R.A. (2011). Isolation from animal tissue and genetic transformation of *Coxiella burnetii* are facilitated by an improved axenic growth medium. *Applied and Environmental Microbiology* 77**,** 3720-3725.

Sales, M., De Freitas, O., Zucoloto, S., Okano, N., Padovan, G., Dos Santos, J., and Greene, L. (1995). Casein, hydrolyzed casein, and amino acids that simulate casein produce the same extent of mucosal adaptation to massive bowel resection in adult rats. *The American Journal of Clinical Nutrition* 62**,** 87-92.

Sandoz, K.M., Beare, P.A., Cockrell, D.C., and Heinzen, R.A. (2016). Complementation of arginine auxotrophy for genetic transformation of *Coxiella burnetii* by use of a defined axenic medium. *Applied and Environmental Microbiology* 82**,** 3042-3051.

Wattam, A.R., Abraham, D., Dalay, O., Disz, T.L., Driscoll, T., Gabbard, J.L., Gillespie, J.J., Gough, R., Hix, D., and Kenyon, R. (2013). PATRIC, the bacterial bioinformatics database and analysis resource. *Nucleic Acids Research***,** gkt1099.
