# Supplementary figures and images for: Multiple Substrate Usage of Coxiella burnetii to Feed a Bipartite Metabolic Network
Source: Front Cell Infect Microbiol. 2017 Jun 29;7:285. doi: 10.3389/fcimb.2017.00285 (PMC5489692; doi:10.3389/fcimb.2017.00285)

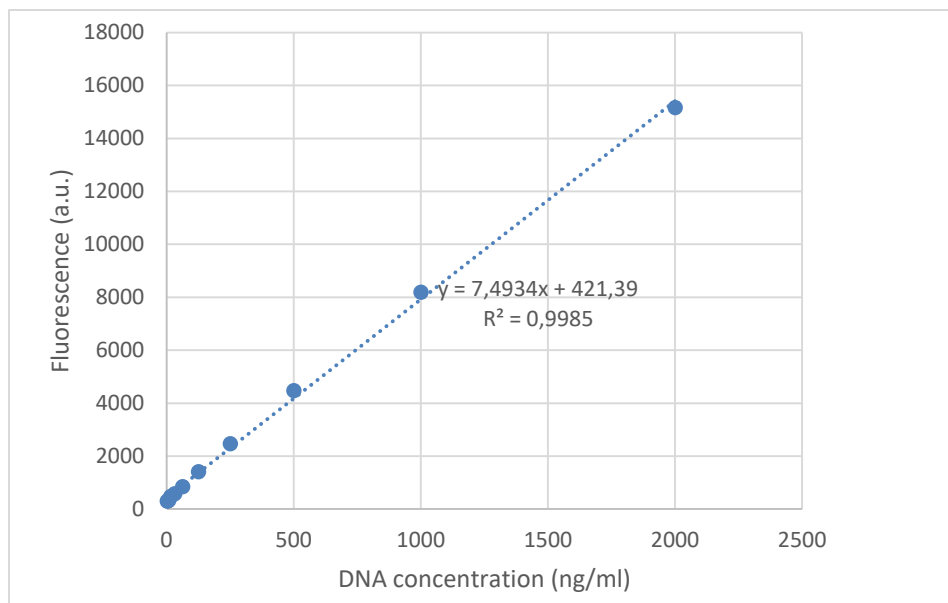

| Fluorescence | ng/ml |
|--------------|-------|
| 15169        | 2000  |
| 8195         | 1000  |
| 4477         | 500   |
| 2472         | 250   |
| 1415         | 125   |
| 850          | 62.5  |
| 582          | 31.25 |
| 481          | 15.6  |
| 332          | 7.8   |
| 316          | 3.9   |
| 305          | 1.95  |

Supplement: Supplementary file 2 [file DataSheet2.PDF]
